# An inter-laboratory comparison of an NLRP3 inflammasome activation assay and dendritic cell maturation assay using a nanostructured lipid carrier and a polymeric nanomedicine, as exemplars

**DOI:** 10.1007/s13346-022-01206-6

**Published:** 2022-07-15

**Authors:** Rob J. Vandebriel, Christopher A. W. David, Jolanda P. Vermeulen, Neill J. Liptrott

**Affiliations:** 1 National Institute for Public Health & the Environment, Bilthoven, the Netherlands; 2 Immunocompatibility Group, Department of Pharmacology and Therapeutics, Institute of Systems, Molecular, and Integrative Biology, University of Liverpool, Liverpool, UK

**Keywords:** NLRP3 inflammasome activation, Monocyte-derived dendritic cell, Dendritic cell maturation, Nanostructured lipid carrier, Polymeric nanomedicine, Inter-laboratory comparison

## Abstract

Nanoparticles including nanomedicines are known to be recognised by and interact with the immune system. As these interactions may result in adverse effects, for safety evaluation, the presence of such interactions needs to be investigated. Nanomedicines in particular should not unintendedly interact with the immune system, since patient’s exposure is not minimised as in the case of ‘environmental’ nanoparticles, and repeated exposure may be required. NLRP3 inflammasome activation and dendritic cell (DC) maturation are two types of immune mechanisms known to be affected by nanoparticles including nanomedicines. NLRP3 inflammasome activation results in production of the pro-inflammatory cytokines IL-1β and IL-18, as well as a specific type of cell death, pyroptosis. Moreover, chronic NLRP3 inflammasome activation has been related to several chronic diseases. Upon maturation, DC activate primary T cells; interference with this process may result in inappropriate activation and skewing of the adaptive immune response. Here, we evaluated the effect of two nanomedicines, representing nanostructured lipid carriers and polymers, on these two assays. Moreover, with a view to possible future standardisation and regulatory application, these assays were subject to an inter-laboratory comparison study using common SOPs. One laboratory performed three independent NLRP3 inflammasome activation experiments, while the other performed a single experiment. Two laboratories each performed three independent DC maturation experiments. While the nanostructured lipid carrier only showed marginal effects, the polymers showed major cytotoxicity. No evidence for inflammasome activation or DC maturation was demonstrated. Intra- and inter-laboratory comparison showed clearly reproducible results.

**Graphical abstract:**

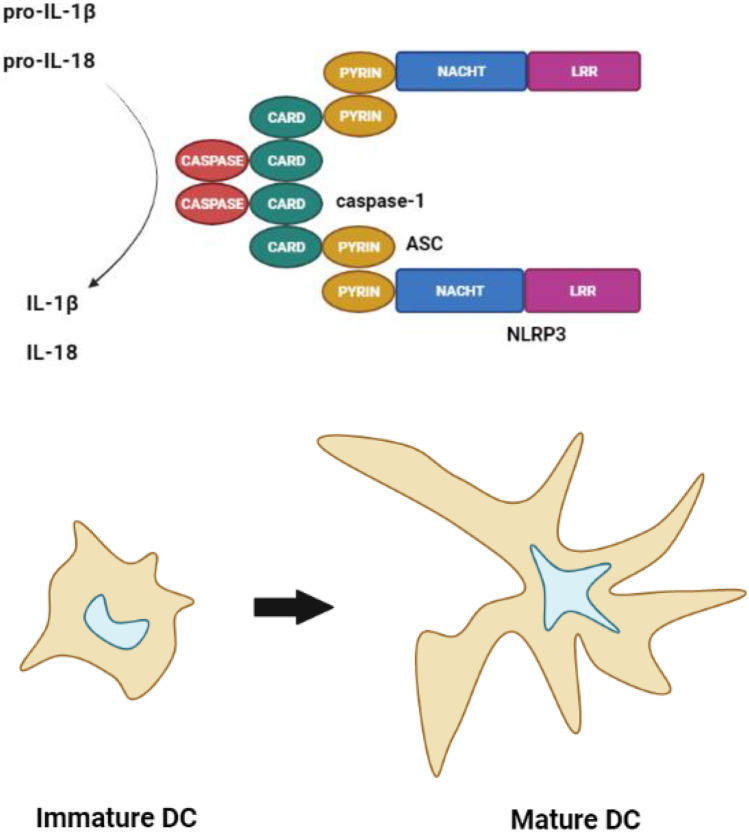

**Supplementary Information:**

The online version contains supplementary material available at 10.1007/s13346-022-01206-6.

## Introduction

Nanoparticles (NP) are known to interact with the immune system [1]. This also holds for nanomedicines [2]. Generally, effects on the immune system can be regarded as detrimental as it disturbs the intricate homeostasis of the system. Especially nanomedicines should not interact with the immune system, since patients are intentionally exposed and often so for a prolonged period. The degree and nature of NP interaction with the immune system depends on the NP’s characteristics [3, 4]. However, the relationship between these is still not completely understood, meaning that prediction of effects on the immune system from these characteristics is limited. A series of (preferably in vitro and high throughput) assays is therefore required, to establish possible effects on the immune system. In a recent analysis, we, among others, listed immune system endpoints for which, however required by regulatory authorities, no generally accepted assays exist [5]. From this list of endpoints, two assays, measuring different immune mechanisms, are the subject of the current study. Both mechanisms are known to be affected by nanomaterials and are linked to adverse immune effects [6–8].

The NLR family, pyrin domain-containing 3 (NLRP3) inflammasome consists of a NLRP3 scaffold, an apoptosis-associated speck-like protein containing a CARD (ASC) adaptor, and pro-caspase-1. Upon activation, NLRP3 recruits ASC. ASC then binds to pro-caspase-1, resulting in auto-cleavage of this pro-enzyme to become the active enzyme caspase-1. Caspase-1 processes pro-IL-1β and pro-IL-18 to bioactive IL-1β and IL-18, respectively [9]. Please refer to this publication also for a clear representation of the pathways involved. These cytokines are potent mediators of inflammation. Next to host-derived molecules and a multitude of infectious agents [9], the NLRP3 inflammasome can be induced by a wide range of xenobiotics including NP [6]. Its activation is associated with various inflammatory diseases, including lung fibrosis, obesity and type-2 diabetes [7].

Dendritic cells (DCs) are sentinel cells that are pivotal in the initiation of adaptive immune responses [10]. Moreover, they integrate various stimuli, such as from different pathogen-associated molecular patterns (PAMPs) and the cytokine milieu. PAMPs are detected by pattern recognition receptors (PRRs) that are highly expressed by DCs. Important classes of PRR form the Toll-like receptors and Nod-like receptors. Importantly, the nature of the immune response following DC maturation is significantly influenced by the PRR (or combination of different PRR). In this way, DCs form an important link between the innate and adaptive immune response. DCs appear as immature DCs that are well capable of ingesting protein antigens and as mature DCs that are especially capable of presenting peptides to naive T cells. This process of DC maturation is central to the functioning of DC. Various types of NP can influence the process of DC maturation and by that immune function [8]. DC maturation can be readily measured by cell surface marker expression and cytokine production. The panel of cell surface markers used to measure DC maturation generally comprises HLA-DR (MHC class II) and the T cell co-receptors CD40, CD80 and CD86, although additions to this panel such as CD83, PD-L1 and DC-SIGN are sometimes included, while in other studies the panel used is more limited. The cytokines measured to evaluate DC maturation are mostly IL-12p40 or IL-12p70, but also IL-10 and TNF-α.

Although DC harbour a fully functional NLRP3 inflammasome, for the NLRP3 inflammasome activation assay, we chose to use macrophages derived from THP-1 human monocytes, since (1) the NLRP3 inflammasome is more strongly expressed in macrophages compared to DC, (2) a monocyte cell line is likely to provide more reproducible data than primary monocytes and (3) there is no possible interference by DC maturation on NLRP3 inflammasome activation. DC obtained from cell lines have limited functionality compared to those obtained from primary monocytes, justifying primary monocytes as a source for the DC maturation assay.

Here, we present the results of an inter-laboratory comparison study of two assays, NLRP3 inflammasome activation (using macrophages derived from THP-1 monocytes), and DC maturation (using DC derived from primary monocytes). The assays were performed by two laboratories using common SOPs. While RIVM performed three independent replicate experiments of the NLRP3 inflammasome activation assay, the University of Liverpool performed one experiment. RIVM and the University of Liverpool each performed the DC maturation assay in three independent replicate experiments. Two types of nanomedicines were tested: the nanostructured lipid carrier LipImage™ 815 and a nanocarrier composed of poly (alkyl cyanoacrylate) polymer. The latter was tested both empty and loaded with Cabazitaxel.

## Materials and methods

### Nanomedicines

Two types of nanomedicines were tested: (i) the nanostructured lipid carrier LipImage™ 815 [11], and (ii) the nanocarrier composed of the poly (alkyl cyanoacrylate) (PACA) polymer: poly (2-ethylbutyl cyanoacrylate) (PEBCA). PEBCA was tested both empty and loaded with Cabazitaxel (CBZ) [12]. In the present paper, these polymer nanocarriers are designated PACA and PACA-CBZ, respectively.

#### LipImage™ 815 synthesis and characterisation

Batches of LipImage™ 815 were prepared by high-pressure homogenization (HPH). The lipid phase comprised 19.125 g of soybean oil, 6.375 g of Suppocire™ NB, 4.875 g of lecithin, and 150 mg of IR-870 oleyl (molar mass: 986.29 g/mol), which was synthetized as previously described [11]. The aqueous phase comprised 25.875 g of Myrj™ S40 and 110 ml NaCl 154 mM. Mixtures of lipid and aqueous phases were pre-emulsified using a mechanical disperser (Ultra-T25 Digital Turrax, IKA) operated at 15,000 rpm for 5 min. The emulsion was then processed with a High-Pressure Homogenizer (Panda Plus 2000, GEA Niro Soavi, Italy) operated for 16 cycles with a total pressure of 1250 bars, the pressure of the second stage chamber being set at 50 bars and the cooling system at 30 °C. Batches of 200 g of particles were then purified by 5 µm filtration followed by tangential flow filtration (Labscale TFF system, Millipore) against NaCl 154 mM through a Pellicon XL Biomax™ cassette (Merck) operated at a trans-membrane pressure of 1 bar at a flow rate of 2 ml/min. The nanoparticle dispersion was adjusted to a concentration of 100 mg/ml and filtered through a 0.22 μm Millipore membrane for sterilization before storage and use.

Dynamic light scattering (DLS) was used to determine the particle hydrodynamic diameter and zeta potential (Zeta Sizer Nano ZS, Malvern Instrument, Orsay, France). Particle dispersions were diluted to 2 mg/ml of lipids in 0.22 µm filtered 0.1 X PBS and transferred in Zeta Sizer Nano cells (Malvern Instrument) before each measurement, performed in triplicate. Results (Z-average diameter, dispersity index, ζ-potential) were expressed as mean and standard deviation of three independent measurements performed at 25 °C. The encapsulation efficiency and payload of IR780-oleyl dye in the LipImage™ 815 were determined by high-performance liquid chromatography (HPLC WATERS Alliance 2695/Fluorescence 2475 detector) and compared with a calibration curve established from the reference fluorophore IR780-Oleyl alone, as previously described [13]. The theoretical amount of IR780-Oleyl encapsulated in a batch of LipImage™ 815 at 100 mg/ml lipid nanoparticles is 266 µM. The size, polydispersity index, ζ-potential and dye loading of LipImage™ 815 is shown in Table 1.

**Table 1:** Size, polydispersity index, ζ-potential, and dye and drug loading of the nanomedicines tested. Characteristics as measured by the producers CEA and SINTEF

	Size (nm)	PDI	ζ-pot (mV)	Drug loading (wt %)	Dye loading (wt %)
LipImage™815	53 ± 1	0.15	− 1.5 ± 1	–	0.35%
PACA	136.2	0.11	− 4.8	–	–
PACA-CBZ	121.8	0.14	− 5.5	10.8%	–

#### PACA synthesis

PACA nanoparticles were synthesized under aseptic conditions at SINTEF (Trondheim, Norway) by mini-emulsion polymerisation. Prior to synthesis, all solutions were sterile filtered, and all equipment was autoclaved. An oil phase consisting of poly(ethyl butyl cyanoacrylate) (PEBCA) (Cuantum Medical Cosmetics) containing 2 wt % Miglyol 812 (Cremer) and 10 wt % vanillin was prepared. For drug-loaded particles, 12 wt % CBZ (Shanghai Biochempartner Co., Ltd. (Shanghai, China)) was added to the oil phase and only 2 wt % vanillin was used. For dye-loaded particles, either 0.4 wt % IR-780-Oleyl (custom synthesis at CEA LETI) or NR668 (modified Nile Red, custom synthesis at SINTEF [14]) was added to the oil phase.

An aqueous phase consisting of 0.1 M HCl containing the two PEG stabilisers (Brij^®^L23 and Kolliphor^®^HS15, both Sigma-Aldrich, 5 wt % of each) was added to the oil phase. The water and oil phases were mixed and immediately sonicated for 3 min on ice (6 × 30 s intervals, 60% amplitude, Branson Ultrasonics digital sonifier). The solution was rotated (15 rpm) at room temperature (RT) overnight. The pH was then adjusted to 5.0 to allow further polymerisation at RT for 5 h. The dispersions were dialyzed (Spectra/Por dialysis membrane MWCO 100.000 Da) against 1 mM HCl to remove unreacted PEG. The size (z-average), polydispersity index (PDI) and the ζ-potential of the NPs in phosphate buffer (10 mM, pH 7.0) were measured by DLS and laser Doppler Micro-electrophoresis using a Zetasizer Nano ZS (Malvern Instruments).

To calculate the amount of encapsulated drug, the drug was extracted from the particles by dissolving them in acetone (1:10), and quantified by liquid chromatography coupled to mass spectrometry (LC–MS/MS) using an Agilent 1290 HPLC system coupled to an Agilent 6490 triple quadrupole mass spectrometer. The size, polydispersity index, ζ-potential and drug loading of PACA and PACA-CBZ is shown in Table 1.

### NLRP3 inflammasome activation

#### Cell line maintenance

THP-1 cells (ATCC TIB-202) were cultured in complete cell culture medium (CCM), that is: RPMI 1640 (Gibco) supplemented with foetal calf serum (10% v/v, Greiner-Bio), penicillin (100 U/ml), and streptomycin (100 µg/ml) (Gibco). The cells were sub-cultured twice per week, seeded to a cell density of 2 × 10^5^ cells/ml, and not allowed to grow to a density beyond 1 × 10^6^ cells/ml. Cells were not cultured for more than twenty passages to prevent genetic divergence.

#### Differentiation of THP-1 cells

The THP-1 cells were differentiated into macrophage-like cells by culturing for 3 h in the presence of 100 ng/ml phorbol 12-myristate 13-acetate (PMA) (Sigma) in 96-well format at a cell density of 5 × 10^5^ cells/ml, 100 µl/well. After this incubation, the cells were adherent. The cells were visually inspected for macrophage-like appearance. The medium was replaced with fresh culture medium without PMA and the plates were incubated for 24 h at standard conditions (humidified incubator at 37 °C, 5% CO_2_). After this incubation period, the cells were exposed to a two-fold dilution series of LipImage™ 815, PACA, or PACA-CBZ (2, 4, 8, 16, 32, 64 and 128 µg/ml), for 48 h at standard conditions. As positive control for NLRP3 inflammasome activation, nigericin (InvivoGen) was used (0.625 and 1.25 µg/ml). CCM was used as negative control. Cells were used for viability testing; culture supernatants were frozen at − 80 °C until further use (ELISA).

#### Viability of THP-1 cells

The viability of the cells after exposure was assessed using the cell proliferation reagent WST-1 (Sigma-Aldrich). Exposed cells (and controls) were incubated for 2.5 h under standard conditions in the presence of 10% (v/v) WST-1 reagent. After incubation, the absorbance (A) was measured in each well at 440 nm (A_440_) and corrected for background absorbance at 620 nm (A_620_). Exposures for viability assessment were performed in triplicate and the viability was calculated as follows: (A (cells in medium, X) – A (medium only, X))/A (cells in medium, C) – A (medium only, C), where X is a specific concentration nanomedicine or positive control and C the CCM control. The viability was expressed as percentage of the control. As a control, for each nanomedicine at the highest exposure concentration (in CCM), the A_440_-A_620_ signal was measured and found not to interfere with the read-out signal of the WST-1 assay.

#### IL-1β ELISA

The IL-1β concentrations in the culture supernatant were determined using ELISA (eBioscience) according to the manufacturer’s instructions. An 8-point, twofold dilution series of a cytokine standard was prepared, diluent was used as blank. A calibration curve was calculated using 5-parameter curve fitting. Exposures for the assessment of IL-1β secretion were performed in four wells per condition. The supernatants were tested in a twofold dilution, except nigericin (0.625 µg/ml in a fivefold dilution and 1.25 µg/ml in a 30-fold dilution) to stay within the standard curve concentration range.

### Concentration–response modelling

Concentration–response modelling for viability and IL-1β production was performed with the statistical software package PROAST [15] (version 70.3) within the software environment ‘R’ [16] (version 4.1.0).

In this approach, a concentration–response dataset is evaluated as a whole by fitting a concentration–response model over the entire concentration range studied. Having fitted a concentration–response model to the data, this curve is used to assess the benchmark concentration (BMC) associated with the benchmark response (BMR) of 50%. The choice of the model for deriving the BMC follows from a procedure of applying likelihood ratio tests to the five members of the following two nested families of models:

$$\begin{array}{ll}\mathrm{Exponential\; family}&\mathrm{Hill\; family}\\\mathrm{E1}\!\!:\mathrm{y=a}&\mathrm{H1}\!\!: \mathrm {y=a}\\\mathrm {E2}\!\!: \mathrm{y=a} \;\mathrm{exp}\; \mathrm{(b\;x)}&\mathrm{H2}\!\!:\mathrm{y=a}\;(1-\mathrm{x}\;/\;\mathrm{(b+x))}\\\mathrm{E3}\!\!:\mathrm{y=a}\;\mathrm {exp} \;\mathrm{(b\;x^{d})}&\mathrm{H3}\!\!:\mathrm{y=a}\;\mathrm{(1-x^{d}\;/\;(b^{d}+x^{d})})\\\mathrm{E4}\!\!:\mathrm{y=a}\;\mathrm{(c-(c-1))\;exp\; (b\;x)}&\mathrm{H4}\!:\mathrm{y=a}\;\mathrm{(1+(c-1)\;x\;/\;(b+x))}\\\mathrm{E5}\!:\mathrm{y=a}\;\mathrm{(c-(c-1))\;exp\;(b\;x^{d})}&\mathrm {H5}\!:\mathrm{y=a}\;\mathrm{(1+(c-1)\;x^{d}\;/\;(b^{d}+x^{d}))}\end{array}$$

where y is any continuous endpoint and x denotes the concentration. In these models, the parameter ‘a’ represents the background response and the parameter ‘b’ can be considered as the parameter reflecting the efficacy of the chemical (or the sensitivity of the subject). First, the likelihood ratio test was used to establish whether extension of a model by increasing the number of parameters resulted in a statistically significant improvement of the fit. The model that could not be significantly improved was considered as the most appropriate member (which adequately fits but does not overfit the data) within each family. In addition, a goodness of fit test (*P* > 0.05) was applied by comparing the log-likelihood of the fitted model to that associated with the so-called full model. The full model simply consists of the observed (mean) responses at each applied concentration. The model is accepted when the log-likelihood value of the fitted model is not significantly worse than that of the full model. Subsequently, the BMCs are derived from the different models and the 90% confidence intervals (CIs) surrounding the BMCs are calculated using the profile-likelihood method. The BMC used in the analysis was the geometric average of the BMCs derived for the different models. The 90% CI surrounding this BMC comprised the BMCL and the BMCU found for the BMC estimates derived from the different models.

The standard operating procedure is included (Supplementary Information S1).

### Dendritic cell maturation

#### Isolation of CD14^+^ cells

Human buffy coats were purchased from the Dutch blood bank (Sanquin, Amsterdam) and obtained the night before isolation of the cells. They were kept at RT until starting cell isolation the next morning. At the University of Liverpool, blood was obtained from healthy volunteers on the day of the experiment. The buffy coat was diluted 1:1 with PBS. Peripheral blood mononuclear cells (PBMCs) were isolated from the buffy coat by centrifugation (1000 g, 30 min, 20 °C) over Ficoll (Lymphoprep; Axis Shield, Oslo, Norway). After washing with PBS, red blood cells were lysed by resuspending the cell pellet in ACK buffer (156 mM NH_4_Cl, 10 mM KHCO_3_, 0.1 mM Na_2_EDTA, pH 7.3) and a subsequent wash with PBS. CD14^+^ cells were positively selected from the cell suspension using a magnetic-activated cell sorting (MACS) kit with CD14-specific antibodies (MACS, Miltenyi Biotec, Leiden, the Netherlands) according to the manufacturer’s instructions. To achieve a high purity, a lower amount of antibody was used than recommended (5 µl per 10^7^ cells). The positive and negative fractions from the MACS were analysed by flow cytometry to determine CD14^+^ purity (see Table 2 for antibody panel).

**Table 2:** Antibody panel for the assessment of CD14.^+^ purity of cell samples by flow cytometry

**Marker**	**Label**	**Dilution**	**Manufacturer**
CD14	PE	1:50	Becton Dickinson
Live/dead	Aqua	1:400	Invitrogen

Staining was done in two consecutive steps. First, the cells were stained with Live/dead (in PBS, 0.2 mM EDTA) at 4 °C for 30 min. Second, the cells were washed with FACS buffer (PBS pH 7.2, 0.2 mM EDTA, 0.5% BSA), and stained with anti-CD14 antibody in FACS buffer at 4 °C for 30 min.

**Fig. 1:**
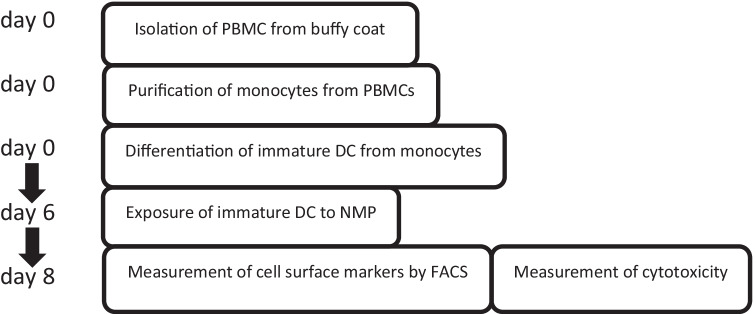
Flow diagram of DC maturation assay

The CD14^+^ cell fraction was resuspended in complete culture medium (CCM): RPMI 1640 GlutaMAX (Gibco), 10% Foetal Calf Serum (FCS; Hyclone; GE Healthcare), 1% pen/strep (Gibco), 450 U/ml GM-CSF (PeproTech) and 350 U/ml IL-4 (Active Bioscience). For a flow diagram, see Fig. 1.

#### Differentiation of CD14^+^ cells to immature DC, and exposure to nanomedicines

The CD14^+^ cells were seeded in 12-well plates, 1 ml/well, 3 × 10^5^ cells/ml and incubated at 37 °C and 5% CO_2_ in a humidified incubator. After 3 days, 100 µl RPMI 1640 GlutaMAX containing 10% FCS, 4500 U/ml GM-CSF and 3500 U/ml IL-4 was added to each well to a final concentration of approximately 450 U/ml GM-CSF and 350 U/ml IL-4. After 6 days, 750 µl culture medium was removed and spun down. The pellet was resuspended in CCM and seeded back into the wells with a dilution series of LipImage™ 815, PACA or PACA-CBZ. LPS (100 ng/ml) and R848 (5 µg/ml) were used as positive controls, 10% PBS as negative control. The plates were placed back in the incubator for 44–48 h until harvest for analysis. For harvesting, from each well the culture medium was collected and spun down. Each supernatant was individually transferred to a fresh tube and stored at – 80 °C for ELISA. In the meantime, cold PBS was put on the cells that were attached to the wells. After detaching the cells by gentle scraping and pipetting, they were collected and added to the tube in which already part of the cells was collected. These cells were divided over two wells for staining with the two separate antibody panels.

#### Flow cytometric analysis of cultured cells

Maturation of the DCs was assessed by flow cytometry (FACS) using two antibody panels (Table 3). In addition, Forward Scatter (FSC; a measure of cell size) and Side Scatter (SSC; a measure of internal complexity (i.e. granularity)) were measured.

**Table 3:** Antibody panels used for the assessment of DC maturation by flow cytometry

**Marker**	**Label**	**Dilution**	**Manufacturer**
Panel 1			
CD80	FITC	1:40	Becton Dickinson
CD14	PE	1:50	Becton Dickinson
PD-L1	APC	1:400	eBioscience
HLA-DR	Pacific Blue	1:1000	Biolegend
Live/dead	Aqua	1:1000	Invitrogen
Panel 2			
CD83	FITC	1:20	Becton Dickinson
CD40	PE	1:10	Becton Dickinson
DC-SIGN	APC	1:200	Becton Dickinson
CD86	Pacific Blue	1:800	Biolegend
Live/dead	Aqua	1:1000	Invitrogen

First, the cells were washed twice with PBS. Second, the cells were stained with Live/dead in PBS, 0.2 mM EDTA at 4 °C for 30 min. Third, the cells were washed once FACS buffer (PBS pH 7.2, 0.2 mM EDTA, 0.5% BSA). To 100 μl of these cells, 100 μl of panel 1 or panel mix 2 (see Table 3) was added. After incubation at 4 °C for 30 min, the cells were washed twice, spun down and included in FACS buffer. Data was acquired using the FACS Canto II (Becton Dickinson Biosciences) using the settings: (1) FSC: 150; SSC: 350; PE: 488 nm laser (blue), 585/42 filter; Aqua: 405 nm laser (violet), 510/50 filter. (2) Sample flow rate 3 µl/s; sample volume 170 µl; mixing volume 70 µl; mixing speed 180 µl; number of mixes 3; washing volume 800 µl. (3) Compensations were set using beads and DC, on a population of 50% living and 50% dead cells. To obtain dead cells, the living cells were heat-shocked.

**Fig. 2:**
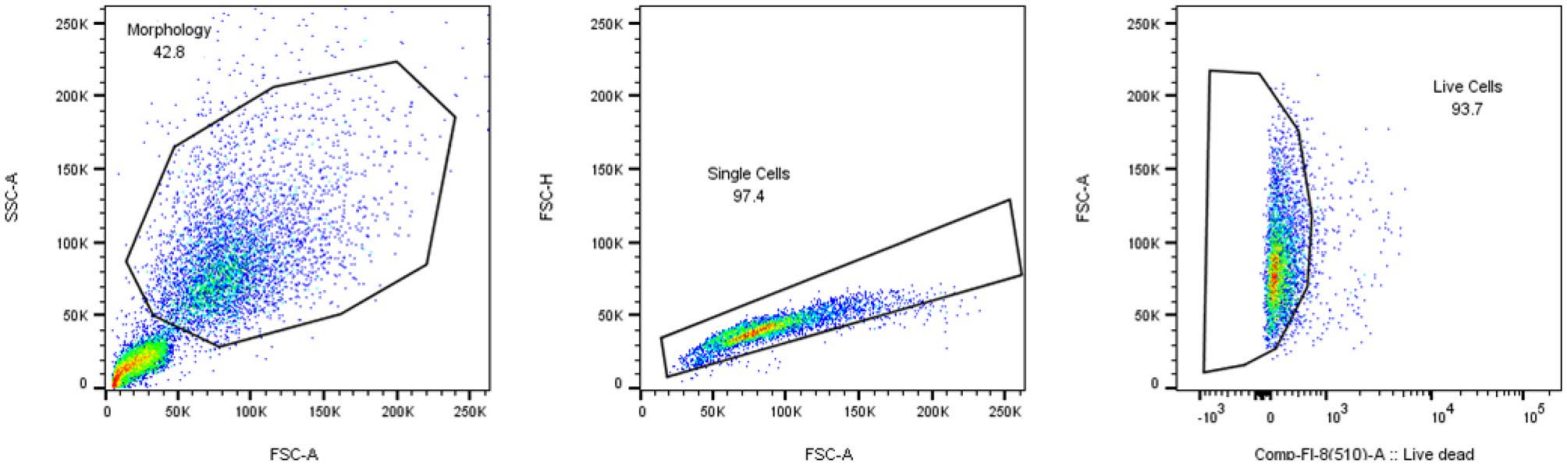
Gating procedure. (1) Gating was done based on the morphology of the cells (left graph). The lower left corner (< 50 K FSC-A (*X*-axis) and < 50 K-SSC-A (*Y*-axis)) is excluded. (2) Within the cell population gated under (1), the single cells were gated (middle graph). In the FSC-A (*X*-axis) vs. FSC-H (*Y*-axis) plot, doublet cells form a population below the diagonal. (3) Within the cell population gated under (2), the live cells were gated (right graph). In the Live/dead staining (*X*-axis) vs. FSC-A (right axis), the dead cells scatter to the right

Data were analysed using FlowJo software (Becton Dickinson). Gating was done according to Fig. 2.

#### Determination of cultured dendritic cell viability

The CD14^+^ cells were seeded in 96-well plates, 200 µl/well, 3 × 10^5^ cells/ml and incubated at 37 °C and 5% CO_2_ in a humidified incubator. The protocol as described above ‘Differentiation of CD14 + cells to immature DC, and exposure to nanomedicines’ section was used (and done concurrently). For viability assessment, the protocol as described above ‘Viability of THP-1 cells’ section was used.

### Concentration–response modelling

The method as described above ‘Concentration–response modelling’ section was used.

The standard operating procedure is included (Supplementary Information S2).

## Results

### Inflammasome activation

### RIVM

**Fig. 3:**
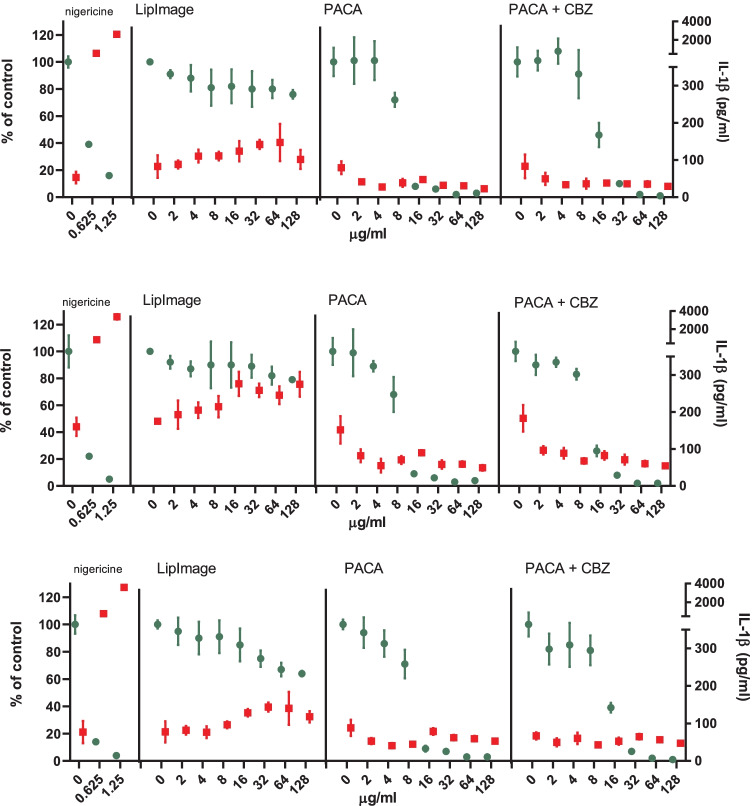
NLRP3 inflammasome activation by LipImage™ 815, PACA, and PACA-CBZ. Nigericin: positive control. Green (plotted to the left Y-axis): viability (percentage of untreated control). Red (plotted to the right *Y*-axis): IL-1β production (pg/ml). Three independent experiments are shown. Mean ± SD, with *N* = 4 replicates per experiment

PMA-activated THP-1 cells were incubated for 48 h with the positive control nigericin at two concentrations (0.625 and 1.25 µg/ml) and a twofold dilution series of LipImage™ 815, PACA and PACA-CBZ (128, 64, 32, 16, 8, 4 and 2 µg/ml, plus a medium control (0)). After this, viability was evaluated using the WST-1 assay and the IL-1β concentration using an ELISA. The results shown are from three independent experiments (Fig. 3).

The positive control for NLRP3 inflammasome activation, nigericin, showed a strong reduction in viability (25% and 8% of the medium control for the low and high concentration, respectively) and a strong increase in IL-1β production (720 and 3200 pg/ml for the low and high concentration, respectively, where the medium control amounted 110 pg/ml). This data of concentration-dependent reduction in viability and concomitant concentration-dependent increase in IL-1β production suggests a proper functioning of the NLRP3 inflammasome activation assay.

Exposure to LipImage™ 815 resulted in a 30% decrease in viability at the highest concentration tested (128 µg/ml). A 50% increase in IL-1β production (from 110 to 165 pg/ml) was seen at the highest concentration tested. Although a decrease in viability and a concomitant increase in IL-1β production is seen, a hallmark of NLRP3 inflammasome activation, the effects observed are too small to suggest that LipImage™ 815 activates the NLRP3 inflammasome.

Exposure to PACA resulted in a clear decrease in viability within a twofold concentration range, from 70% viability at 8 µg/ml to 9% viability at 16 µg/ml. At these same concentrations, only a small increase in IL-1β production was seen, from 50 to 70 pg/ml. Exposure to PACA-CBZ also resulted in a clear decrease in viability, albeit within a fourfold concentration range, from 85% viability at 8 µg/ml, 37% viability at 16 µg/ml, to 8% viability at 32 µg/ml. At these same concentrations, only a small increase in IL-1β production was seen, being 50 pg/ml at 8 µg/ml, and 60 pg/ml at 16 µg/ml and 32 µg/ml. This data suggests a strong cytotoxic effect of PACA, both with and without CBZ, and no evidence for NLRP3 inflammasome activation.

Comparison of the results between the individual experiments shows a high reproducibility, with some quantitative differences in IL-1β production throughout individual concentration–response curves, but a highly similar shape of both the viability and the IL-1β production concentration–response curves.

Inflammasome activation was also assessed using concentration–response modelling. The three experiments presented in Fig. 3 were analysed (together). Since ISO [17] takes 70% viability as a threshold for cytotoxicity, 30% reduction was chosen as effect size. The concentration at which a 30% effect is obtained is designated here as the effective concentration (EC)_30_. Since no guidance exists on an effect size for markers of NLRP3 inflammasome activation, by default we chose a 30% effect (in this case an increase), similar in size to viability. Next to establishing the EC, the software tool PROAST provides a 90% confidence interval (CI) around a specific EC (here EC_30_). In Table 4, the EC_30_ values and corresponding 90% CIs are shown.

**Table 4:** EC_30_ and 90% CI values of viability (µg/ml)

	**viability**	
	**EC**_**30**_	**90% CI**
PACA	8.15	6.31–10.1
PACA-CBZ	8.00	6.41–9.83

When an EC_30_ could not be calculated (LipImage™ 815: viability; PACA-CBZ: IL-1β production), or the ratio between the upper (95%) and lower (5%) limit around the EC_30_ was > 5 (LipImage™ 815: IL-1β production; PACA: IL-1β production), the data were not considered. The data in Table 3 show that the effects of PACA and PACA-CBZ on viability are highly similar, suggesting that in this assay the reduced viability is only due to PACA and not to CBZ.

### University of liverpool

Exposure to LipImage™ 815 resulted in a 26% increase at the highest concentration tested (128 µg/ml). A 10% decrease in IL-1β production was seen at the highest concentration tested (from 50 to 45 pg/ml). The effects observed do not suggest that LipImage™ 815 activates the NLRP3 inflammasome.

Exposure to PACA resulted in a clear decrease in viability within a twofold concentration range, from 79% viability at 8 µg/ml to 19% viability at 16 µg/ml, so at the same concentrations as seen in the experiments performed at RIVM. At these same concentrations, no effect on IL-1β production (48 pg/ml) was seen. Exposure to PACA-CBZ also resulted in a decrease in viability, albeit within a fourfold concentration range, from 97% viability at 8 µg/ml, 78% viability at 16 µg/ml, to 44% viability at 32 µg/ml. Thus, similar to the findings at RIVM, PACA-CBZ showed a more gradual decrease in viability compared to PACA alone. At these same concentrations, no effect on IL-1β production (48 pg/ml for PACA and 45 pg/ml for PACA-CBZ) was seen. This data suggests a strong cytotoxic effect of PACA, both with and without CBZ, and no evidence for NLRP3 inflammasome activation.

**Fig. 4:**
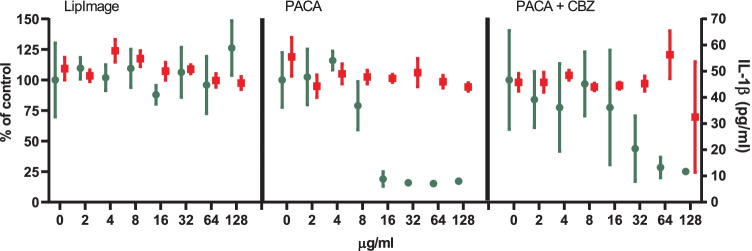
NLRP3 inflammasome activation by LipImage™ 815, PACA, and PACA-CBZ. Green (plotted to the left *Y*-axis): viability (percentage of untreated control). Red (plotted to the right *Y*-axis): IL-1β production (pg/ml). Mean ± SD, with *N* = 4 replicates per experiment

Data from RIVM (Supplementary Information S3A) and the University of Liverpool (Supplementary Information S3B) is included (Fig. 4).

### Dendritic cell maturation

### RIVM

**Fig. 5:**
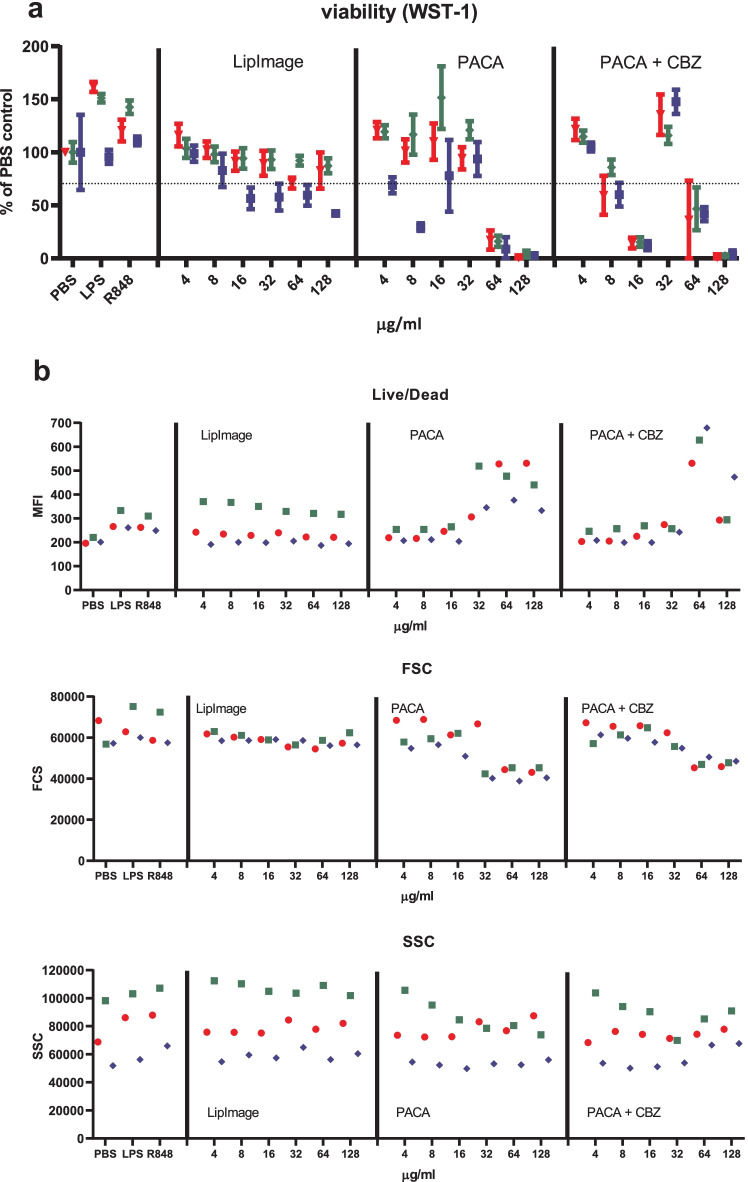

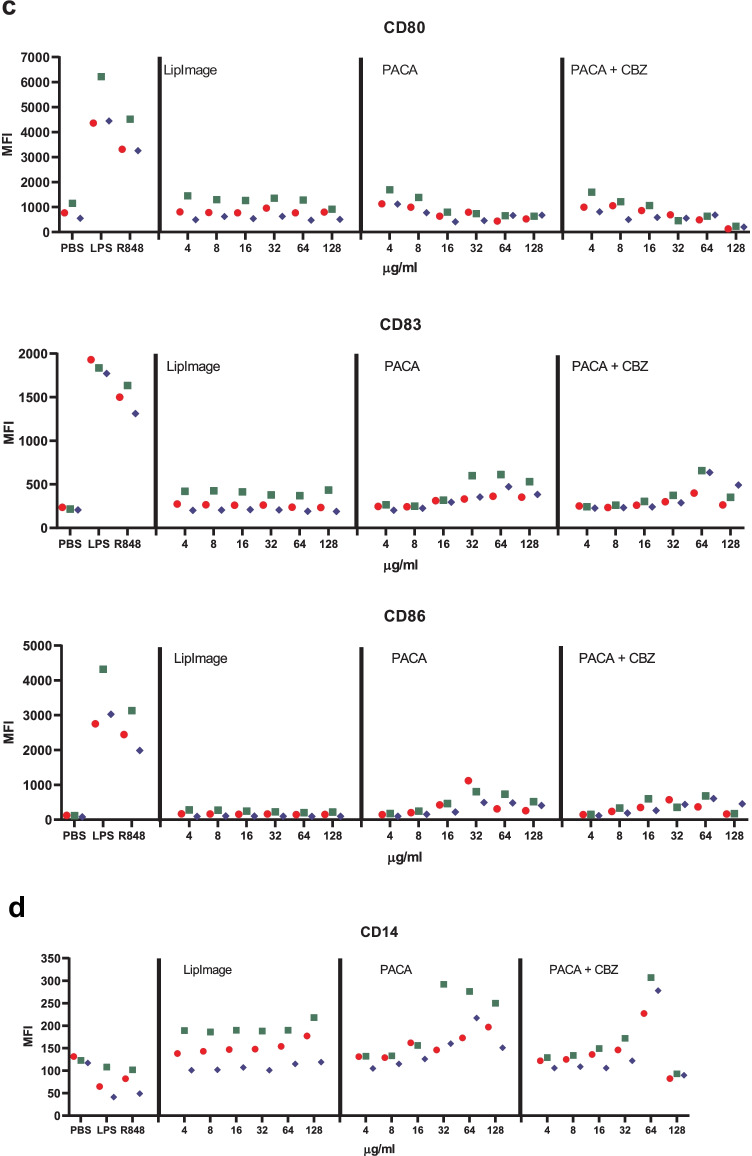

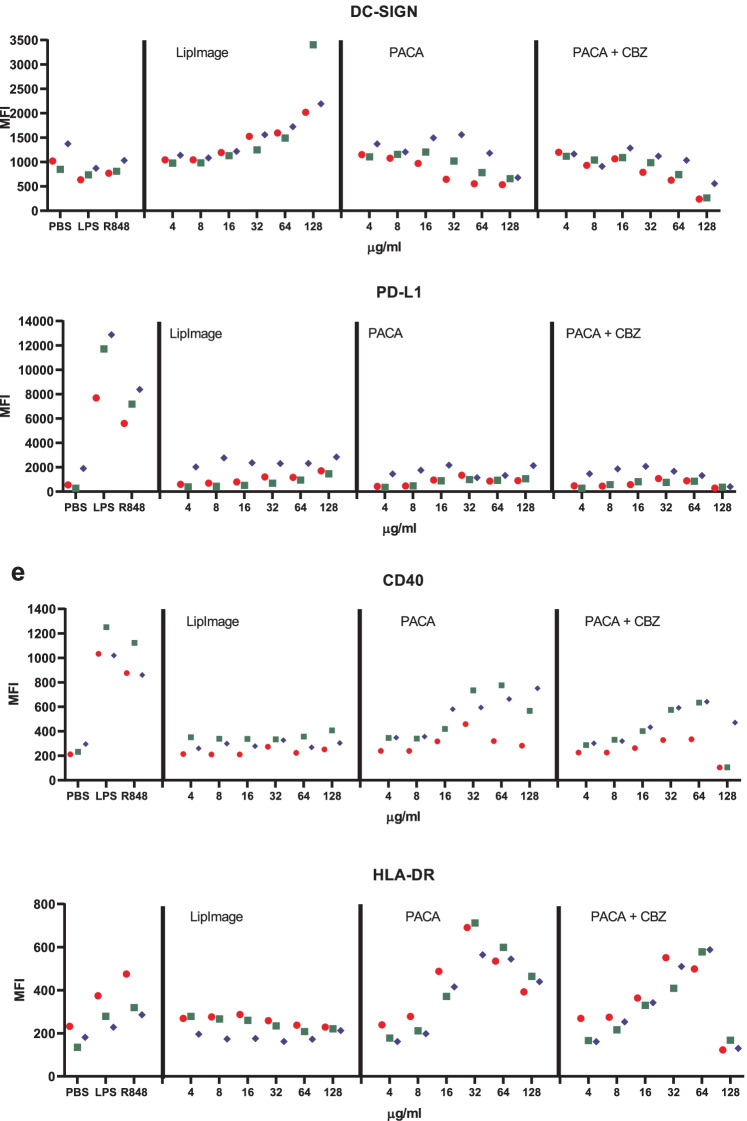
**a** Viability of DC after incubation with LipImage™ 815, PACA, and PACA-CBZ. Red, blue, green: three independent experiments. LPS and R848 were used as positive controls for DC maturation. **b** Effects of incubation with LipImage™ 815, PACA, and PACA-CBZ on Live/dead staining, FSC, and SSC. Red, blue, green: three independent experiments. LPS and R848 were used as positive controls for DC maturation. *MFI* mean fluorescence intensity. **c** Effects of incubation with LipImage™ 815, PACA, and PACA-CBZ on CD80, CD83, and CD86 surface marker expression. Red, blue, green: three independent experiments. LPS and R848 were used as positive controls for DC maturation. *MFI* mean fluorescence intensity. **d** Effects of incubation with LipImage™ 815, PACA, and PACA-CBZ on CD14, DC-SIGN, and PD-L1 surface marker expression. Red, blue, green: three independent experiments. LPS and R848 were used as positive controls for DC maturation. *MFI* mean fluorescence intensity. **e** Effects of incubation with LipImage™ 815, PACA, and PACA-CBZ on CD40 and HLA-DR surface marker expression. Red, blue, green: three independent experiments. LPS and R848 were used as positive controls for DC maturation. *MFI*, mean fluorescence intensity

Monocytes were isolated from buffy coats and differentiated to immature DC. These were incubated for 48 h with a twofold dilution series of LipImage™ 815, PACA and PACA-CBZ (128, 64, 32, 16, 8 and 4 µg/ml, plus a medium control (0)). After this, viability was evaluated using the WST-1 assay and surface marker expression using a FACS. The results shown are from three independent experiments (Fig. 5).

#### Viability

The positive controls for DC maturation, LPS and R848, did not affect viability. Incubation with LipImage™ 815 marginally affected cell viability, averaging 70% at the highest concentration tested (128 µg/ml). PACA showed a clear decrease in viability within a fourfold concentration range, from 103% at 32 µg/ml, 14% at 64 µg/ml and 3% at 128 µg/ml. PACA-CBZ, curiously, showed a biphasic viability curve, averaging 114%, 69%, 14%, 133%, 42% and 2% for the entire concentration range. It should be noted that the results are obtained from three independent experiments using DCs cultured from monocytes of different donors, performed on different weeks. A complete loss of viability at 16 µg/ml does not fit the viability seen for PACA and PACA-CBZ as evaluated by Live/dead staining (see below), or for PACA and PACA-CBZ in the experiments performed by the University of Liverpool (see below). However, in a study dedicated to evaluate the cytotoxicity of LipImage™ 815, PACA and PACA-CBZ, in four different cell lines using two different viability assays, both PACA and PACA-CBZ showed a clear reduction in viability from 2 µg/ml, depending on the cell line and the assay [26].

#### Live/dead, FSC and SSC

The positive controls for DC maturation, LPS and R848, had a minor effect on Live/dead staining, and did not affect FSC (a measure of cell size) and SSC (a measure of internal complexity (i.e. granularity)). No exposure effects of LipImage™ 815 on Live/dead staining, FSC and SSC were seen. PACA and PACA-CBZ induced a clear increase in Live/dead staining from 32 µg/ml and from 64 µg/ml, respectively. PACA and PACA-CBZ decreased FSC from 32 and 64 µg/ml, respectively. PACA and PACA-CBZ did not affect SSC.

#### CD80, CD83 and CD86

The positive controls LPS and R848 clearly induced CD80, CD83 and CD86 expression (5- and 3.5-fold for CD80; 7.5- and sixfold for CD83; 30- and 23-fold for CD86, for LPS and R848, respectively), strongly suggestive of DC maturation. LipImage™ 815, PACA and PACA-CBZ failed to do so, suggesting that none of the three nanomedicines induced DC maturation.

#### CD14, DC-SIGN and PD-L1

LPS and R848 rather similarly downregulated CD14 expression, by 40%. CD14 downregulation by LPS is in line with previous data showing combined endocytosis of LPS, TLR4 and CD14 [18]. DC-SIGN expression was reduced by 30% and 20% by LPS and R848, respectively. PD-L1 expression was induced 11-fold and sevenfold by LPS and R848, respectively. Decreased DC-SIGN expression and increased PD-L1 expression both suggest DC maturation. DC-SIGN is typically downregulated in DC upon maturation [19]. PD-L1 is upregulated in DC upon maturation [20].

LipImage™ 815 did not affect CD14 expression. For PACA and PACA-CBZ, CD14 expression is increased from 32 to 64 µg/ml, respectively. LipImage™ 815 slightly increased DC-SIGN expression at the highest concentration tested (128 µg/ml), while PACA and PACA-CBZ slightly decreased DC-SIGN expression at this concentration. LipImage™ 815, PACA and PACA-CBZ did not affect PD-L1 expression, in line with a lack of effect on the maturation markers CD80, CD83 and CD86.

#### CD40 and HLA-DR

The positive controls LPS and R848 clearly induced CD40 expression (3.5- and threefold, respectively) and to a lesser extent HLA-DR expression (1.6- and twofold, respectively), suggestive of DC maturation. LipImage™ 815 failed to induce expression of either CD40 or HLA-DR. PACA and PACA-CBZ induced a twofold CD40 expression from 32 to 64 µg/ml, respectively, and a twofold HLA-DR expression from 16 to 32 µg/ml, respectively.

We analysed DC maturation also by concentration–response modelling, using the PROAST software tool. The three experiments presented in Fig. 5 were analysed (together). Since ISO [17] takes 70% viability as a threshold for cytotoxicity, 30% was chosen as effect size. The concentration at which a 30% effect is obtained is designated here as the Effective Concentration (EC)_30_. Since no guidance exists on an effect size for markers of DC maturation, by default we chose a 30% effect, similar to viability. Next to establishing the ED, the software tool PROAST allows for generation of a 90% confidence interval (CI) around a specific ED (here EC_30_). In Table 5, the EC_30_ values and corresponding 90% CIs are shown.

**Table 5:** EC_30_ and 90% CI values (µg/ml)

	**PACA**		**PACA-CBZ**	
	**EC**_**30**_	**90% CI**	**EC**_**30**_	**90% CI**
WST-1	43.1	28.7–46.8	63.7	34.1–84.6
Live/dead	24.8	15.3–29.7	36.2	25.7–46.3

When an EC_30_ could not be calculated (LipImage™ 815: all parameters except DC-SIGN and PD-L1; PACA: FSC; PACA-CBZ: CD14, CD40), or the ratio between the upper (95%) and lower (5%) limit around the EC_30_ was > 5 (LipImage™ 815: DC-SIGN and PD-L1; PACA: all parameters except FSC, WST-1 and Live/dead; PACA-CBZ: all parameters except WST-1, Live/Dead, CD14 and CD40), the data were not considered. For WST-1 and Live/dead staining (Table 4), the EC_30_ values for PACA-CBZ were 1.5 times higher than for PACA, possibly suggesting that in these assays PACA-CBZ may be slightly less cytotoxic compared to PACA.

### University of liverpool

**Fig. 6:**
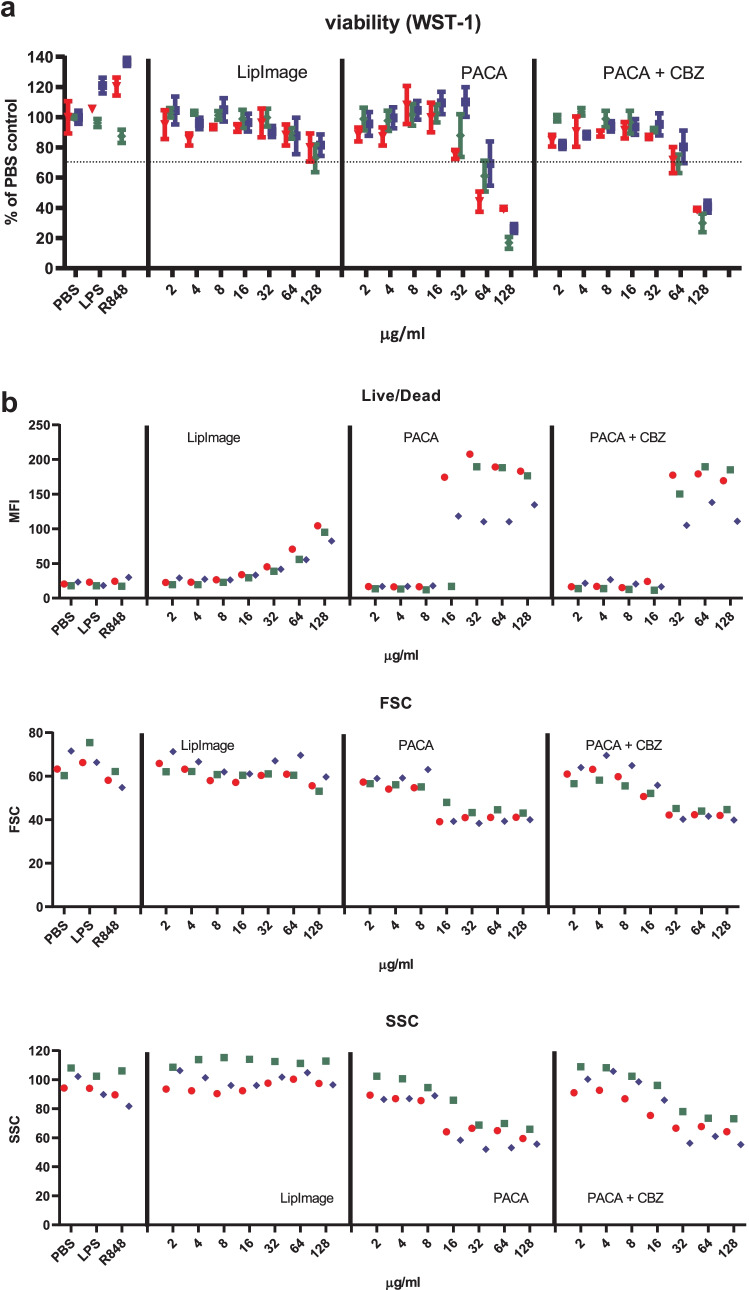

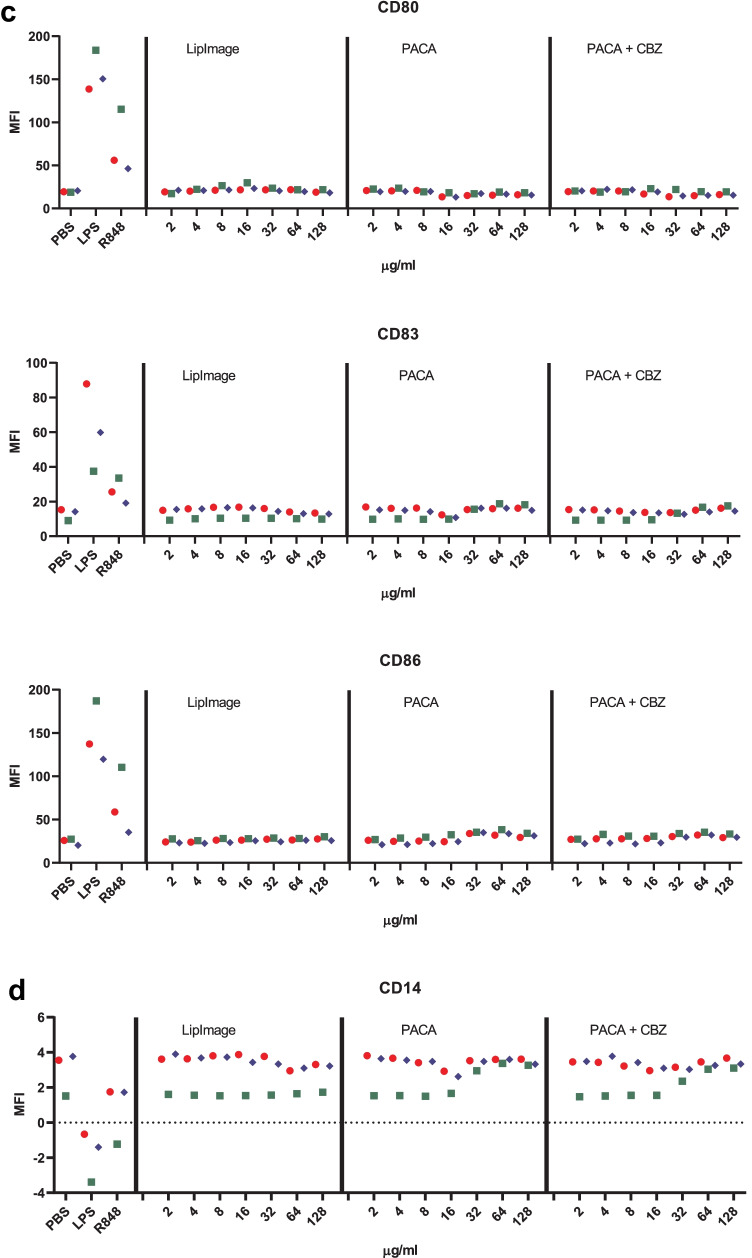

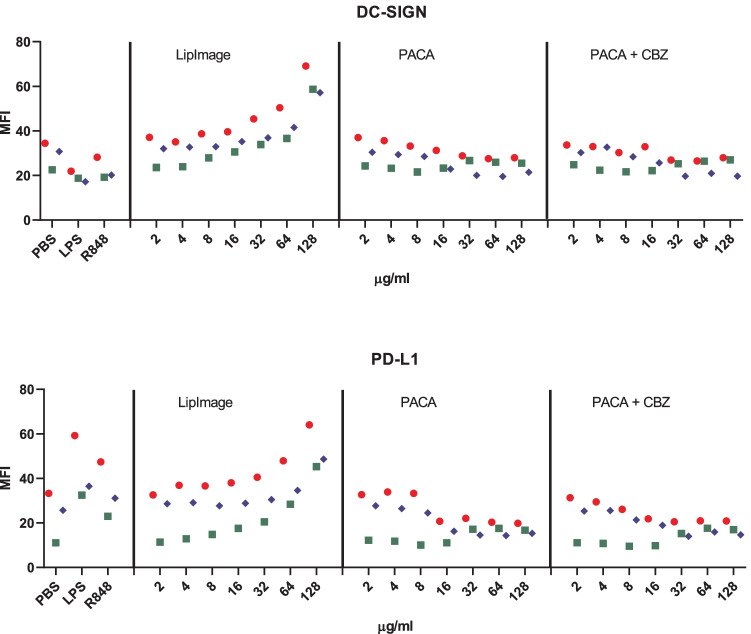
**a** Viability of DC after incubation with LipImage™ 815, PACA, and PACA-CBZ. Red, blue, green: three independent experiments. LPS and R848 were used as positive controls for DC maturation. **b** Effects of incubation with LipImage™ 815, PACA, and PACA-CBZ on Live/dead staining, FSC, and SSC. Red, blue, green: three independent experiments. LPS and R848 were used as positive controls for DC maturation. *MFI* mean fluorescence intensity. **c** Effects of incubation with LipImage™ 815, PACA, and PACA-CBZ on CD80, CD83, and CD86 surface marker expression. Red, blue, green: three independent experiments. LPS and R848 were used as positive controls for DC maturation. *MFI* mean fluorescence intensity. **d** Effects of incubation with LipImage™ 815, PACA, and PACA-CBZ on CD14, DC-SIGN, and PD-L1 surface marker expression. Red, blue, green: three independent experiments. LPS and R848 were used as positive controls for DC maturation. *MFI* mean fluorescence intensity

Monocytes were isolated from buffy coats and differentiated to immature DC. They were incubated for 48 h with a twofold dilution series of LipImage™ 815, PACA, and PACA-CBZ (128, 64, 32, 16, 8, 4 and 2 µg/ml, plus a medium control (0)). After that, viability was evaluated using the WST-1 assay and surface marker expression using a FACS. The results shown are from three independent experiments (Fig. 6).

#### Viability

The positive controls for DC maturation, LPS and R848, did not affect viability. Incubation with LipImage™ 815 marginally affected cell viability, averaging 80% at the highest concentration tested (128 µg/ml). PACA showed a clear concentration-dependent decrease in viability within a fourfold concentration range, from 91% at 32 µg/ml, 58% at 64 µg/ml, to 27% at 128 µg/ml. PACA-CBZ showed a similar decrease in viability, from 91% at 32 µg/ml, 74% at 64 µg/ml and 37% at 128 µg/ml.

#### Live/dead, FSC and SSC

The positive controls for DC maturation, LPS and R848, did not affect Live/dead staining, FSC and SSC. LipImage™ 815 induced a concentration-dependent increase in Live/dead staining, while no exposure effects on FSC and SSC were seen. PACA induced a clear increase in Live/dead staining from 16 µg/ml in two out of three independent experiments. PACA-CBZ induced a clear increase in Live/dead staining from 32 µg/ml. PACA decreased FSC and SSC from 16 µg/ml, while PACA-CBZ affected FSC and SSC from 32 µg/ml.

#### CD80, CD83 and CD86

While LPS and R848 clearly induced CD80, CD83 and CD86 expression showing DC maturation, LipImage™ 815, PACA and PACA-CBZ failed to do so. This suggests that none of the three nanomedicines induced DC maturation.

#### CD14, DC-SIGN and PD-L1

LPS clearly downregulated CD14 expression, in line with previous data showing combined endocytosis of LPS, TLR4 and CD14 [18]. R848 decreased CD14 to a lesser extent than LPS did. DC-SIGN expression was not affected by LPS or R848. PD-L1 expression was induced by LPS and R848.

LipImage™ 815 did not affect CD14 expression. For one of the three independent experiments, for both PACA and PACA-CBZ, CD14 expression is increased from 32 µg/ml. LipImage™ 815 induced the expression of DC-SIGN and PD-L1 from 64 µg/ml. DC-SIGN is typically downregulated in DC upon maturation [19]. No effects on DC-SIGN and PD-L1 expression by PACA and PACA-CBZ were seen.

We analysed DC maturation also by concentration–response modelling. The three experiments presented in Fig. 6 were analysed (together). When an EC_30_ could not be calculated (LipImage™ 815: WST-1, SSC and CD14; PACA: FSC and PD-L1; PACA-CBZ: FSC, CD14, DC-SIGN and PD-L1), or the ratio between the upper (95%) and lower (5%) limit around the EC_30_ was > 5 (LipImage™ 815: Live/dead, DC-SIGN and PD-L1; PACA: CD14), the data were not considered (CD80, CD83 and CD86 were not included). For WST-1, Live/dead staining, and SSC (Table 6), the EC_30_ values for PACA-CBZ were on average 1.5 times higher than for PACA, possibly suggesting that in these assays PACA-CBZ may be slightly less cytotoxic compared to PACA.

**Table 6:** EC_30_ and 90% CI values (µg/ml)

	**PACA**		**PACA-CBZ**	
	**EC**_**30**_	**90% CI**	**EC**_**30**_	**90% CI**
WST-1	51.0	35.0–67.7	72.3	66.3–88.8
Live/dead	10.3	5.34–11.6	17.5	15.2–18.9
SSC	17.5	13.0–17.9	25.9	18.1–35.0

### Inter-laboratory variance in DC parameters

**Fig. 7:**
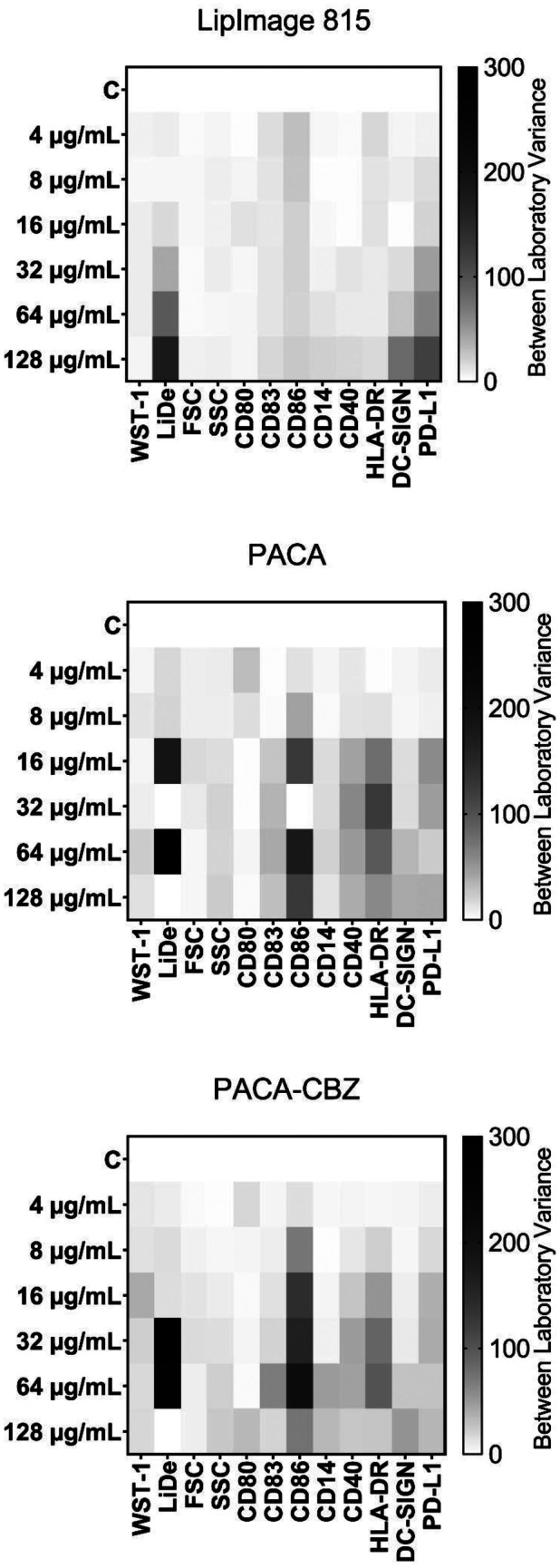
Heat maps of the inter-laboratory variance for each of the three pharmaceuticals tested. For each of *N* = 3 independent experiments, the CCM control (C) was set at 100% and the mean and standard deviation was calculated for these experiments. This was done for each laboratory, after which the variance between the two laboratories was calculated and expressed in a heat map on a scale of 0–300

To evaluate the inter-laboratory variance in all DC parameters including WST-1, we first normalized for each experiment the CCM control to 100%. From this, for each of the three pharmaceuticals, for each of the two partners, and for each individual concentration we calculated the mean and standard deviation over the three independent replicate experiments. After this, the inter-laboratory variance was calculated and expressed in a heat map (Fig. 7). For LipImage™ 815, the largest inter-laboratory variance was for Live/dead staining and, to a lesser extent, DC-SIGN and PD-L1. It should be mentioned that a larger inter-laboratory variance is to be expected when a concentration-dependent effect is seen. For LipImage™ 815, this is seen for DC-SIGN, PD-L1 (for University of Liverpool but not RIVM), but not for Live/dead staining. For PACA and PACA-CBZ, the largest inter-laboratory variance was for Live/dead staining and, to a lesser extent, CD86 and HLA-DR. Of notice, while both WST-1 and Live/dead staining show a rather similar concentration–response as evidenced by concentration–response modelling, the inter-laboratory variance of Live/dead staining is much higher, suggesting that this parameter is much more sensitive to differences between laboratories.

Data from RIVM (Supplementary Information S4A) and the University of Liverpool (Supplementary Information S4B) is included.

## Discussion

Here, we evaluated the effects of two nanomedicines, representing nanostructured lipid carriers and polymers, on two in vitro assays. These assays, NLRP3 inflammasome activation and DC maturation, are among the ones listed to fulfil the information requirements for regulatory acceptance of nano-pharmaceuticals but are still remote from being a standardised assay [5]. Moreover, with a view to possible future standardisation and regulatory application, these assays were subject to an inter-laboratory comparison study, using common SOPs. To this end, one laboratory performed three independent NLRP3 inflammasome activation experiments, while the other performed a single experiment. Two laboratories each performed three independent DC maturation experiments. While the nanostructured lipid nanocarrier only showed marginal effects, the polymers showed major cytotoxicity. No evidence for NLRP3 inflammasome activation or DC maturation was demonstrated. Intra- and inter-laboratory comparison showed clearly reproducible results.

NLRP3 inflammasome activation evaluated by RIVM showed a slight decrease in viability upon exposure to LipImage™ 815 and a sharp decrease in viability upon exposure to PACA and PACA-CBZ. This observation is underlined by concentration–response modelling that showed concentration-dependent cytotoxicity for PACA and PACA-CBZ but not LipImage™ 815. Next, this modelling showed a similar EC_30_ for PACA and PACA-CBZ suggesting that the cytotoxicity observed is caused by PACA and not CBZ. Moreover, the data show a high intra-laboratory reproducibility. Evaluation by the University of Liverpool showed similar results to those obtained by RIVM. No effect on IL-1β production was seen in either laboratory. This suggests that neither of the nanomedicines induced NLRP3 inflammasome activation. In any case, intra- and inter-laboratory reproducibility seems to warrant subsequent steps to standardisation of the assay. In such future studies, nanoparticles well-known to activate the NLRP3 inflammasome should be included, such as SiO_2_ nanoparticles [21].

Measuring only IL-1β (with or without IL-18) may be too limited to establish NLRP3 inflammasome activation. We propose to also include measurement of (1) caspase-1 activity, to better connect IL-1β secretion to NLRP3 inflammasome activation, and (2) caspase-3 activity, to discriminate between pyroptosis resulting from NLRP3 inflammasome activation, and apoptosis as mechanism of cell death.

In a separate inter-laboratory comparison study within the REFINE project, the cytotoxicity of the same batches of the three materials LipImage™815, PACA and PACA-CBZ was tested in four different cell lines using both the WST-8 and the LDH release assay. LipImage™ 815 was non-cytotoxic up to a concentration of 128 µg/ml, whereas PACA caused dose-dependent cytotoxic effects starting from 8 µg/ml. PACA-CBZ showed a less pronounced dose-dependent effect with the lowest concentration of 2 µg/ml causing cytotoxic effects [26].

Outside the inter-laboratory comparison study presented here, but within the REFINE project and using the same SOP as in the study presented here, NLRP3 inflammasome activation by a commercially available liposome (Avanti, Birmingham, AL) was evaluated. The REFINE partners RIVM and CEA each performed 3 independent replicate experiments. Both partners did not observe effects on viability and IL-1β production over the entire concentration range tested (up to of 128 µg/ml) with an appropriate response by the positive control nigericin (Supplementary Information S5). This data suggests a lack of NLRP3 inflammasome activation by the liposome.

The DC maturation assays performed by RIVM showed no effect on viability of LipImage™ 815, whereas a clear decrease in viability by PACA and PACA-CBZ was seen. These findings are underlined by concentration–response modelling that showed concentration-dependent cytotoxicity for PACA and PACA-CBZ but not LipImage™ 815. Next, this modelling showed a 1.5-fold lower EC_30_ for PACA compared to PACA-CBZ, suggesting that the cytotoxicity observed is caused by PACA and not CBZ. These findings were similar for the WST-1 assay and Live/dead-staining, two orthogonal methods to assess cell viability. DC maturation evaluated by the University of Liverpool showed similar results: cytotoxicity induced by PACA and PACA-CBZ but not LipImage™ 815, a 1.5-fold lower EC_30_ for PACA compared to PACA-CBZ, and similar results for the WST-1 assay and Live/dead-staining. It should be noted, however, that for Live/dead staining, the EC_30_ values themselves were rather different between RIVM and the University of Liverpool. For PACA and PACA-CBZ, RIVM established as the most sensitive parameter an increase in HLA-DR, seen from 16 and 32 µg/ml, respectively. The University of Liverpool established a decrease in FSC and SSC as most sensitive parameters, seen from 16 and 32 µg/ml, respectively. Although CD40 and HLA-DR are regarded as DC maturation markers, this data, especially the lack of effect on CD80, CD83 and CD86 expression, suggests that neither of the nanomedicines induce DC maturation. In any case, intra- and inter-laboratory reproducibility seems to warrant subsequent steps to standardisation of the assay. In such future studies, nanoparticles well-known to induce DC maturation should be included, such as TiO_2_ nanoparticles [22].

The heat map shows a considerable difference in Live/dead staining between the two participating laboratories, whereas for WST-1 this difference was limited. This suggests that currently, the WST-1 assay, being the only assay not included in the flow cytometry measurement, should remain to be included in the evaluation of effects on DC maturation.

Flow cytometry is a powerful method for immune cell phenotyping. It is routinely used in clinical immunology laboratories around the world. Moreover, OECD guidelines and ISO standards include the use of flow cytometry, such as the h-CLAT [23]. Still, some of the flow cytometry characteristics may hamper acceptance by regulatory authorities of assays that rely on this method. After data collection, compensations are required to correct for the overlap between adjacent emission spectra of different fluorochromes. Next, to select a specific population of cells serial gating is required, which is done by visual inspection of 2D scatterplots. Both compensation and serial gating are often done manually and may differ between operators. Especially manual gating is subjective, not only because gate setting can be more or less strict, but also the sequence of gating to arrive at the desired cell population may differ [24]. A promising way out is the use of computational flow cytometry, reviewed by Saeys et al. [24] and more recently by Lucchesi et al. [25].

## Conclusions

An inter-laboratory comparison study was performed for two assays, NLRP3 inflammasome activation and DC maturation, using two nanomedicines, the nanostructured lipid carrier LipImage™ 815 and the polymer PACA, either loaded or not with CBZ. PACA and PACA-CBZ showed clear cytotoxicity, whereas LipImage™ 815 did not. Neither of the nanomedicines induced NLRP3 inflammasome activation or DC maturation. Intra- and interlaboratory reproducibility seem to warrant subsequent steps to standardisation of these assays. In such future studies, nanoparticles well-known to activate the NLRP3 inflammasome resp. induce DC maturation should be included.

## Supplementary Information

Below is the link to the electronic supplementary material.

Supplementary file1 (DOCX 10878 KB)

Supplementary file2 (DOCX 211 KB)

Supplementary file3 (XLSX 18 KB)

Supplementary file4 (XLSX 14 KB)

Supplementary file5 (XLSX 28 KB)

Supplementary file6 (XLSX 30 KB)

Supplementary file7 (XLSX 24 KB)

## Data Availability

Data are available upon request to the corresponding author (Rob J. Vandebriel, rob.vandebriel@rivm.nl).
