# Supplementary material for: An inter-laboratory comparison of an NLRP3 inflammasome activation assay and dendritic cell maturation assay using a nanostructured lipid carrier and a polymeric nanomedicine, as exemplars
Source: Drug Deliv Transl Res. 2022 Jul 15;12(9):2225–42. doi: 10.1007/s13346-022-01206-6 (PMC9360168; doi:10.1007/s13346-022-01206-6)
Supplement: Supplementary file 1 — Supplementary file1 (DOCX 10878 KB) [file 13346_2022_1206_MOESM1_ESM.docx]

| Project: | 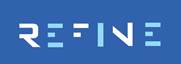 |
| --- | --- |
| NLRP3 inflammasome activation by nanomedicinal products | |
| Subtitle | |

| AUTHORED BY: | DATE: |
| --- | --- |
| Rob Vandebriel, PhD | April 25, 2018 |

| REVIEWED BY: | DATE: |
| --- | --- |
|  |  |
|  |  |

| APPROVED BY: | DATE: |
| --- | --- |
|  | DD/MM/YYYY |

DOCUMENT HISTORY

| Effective Date | Date Revision Required | Supersedes |
| --- | --- | --- |
| DD/MM/YYYY | DD/MM/YYYY | DD/MM/YYYY |

| Version | Approval Date | Description of the Change | | Author / Changed by |
| --- | --- | --- | --- | --- |
| 1.0 | DD/MM/YYYY | All | Initial Document | Name |

Table of Content

[1 Introduction 3](#_Toc507139697)

[2 Principle of the Method 3](#_Toc507139698)

[3 Applicability and Limitations (Scope) 3](#_Toc507139699)

[4 Related Documents 3](#_Toc507139700)

[5 Equipment and Reagents 3](#_Toc507139701)

[5.1 Equipment 3](#_Toc507139702)

[5.2 Reagents 4](#_Toc507139703)

[5.3 Reagent Preparation 4](#_Toc507139704)

[6 Procedure 5](#_Toc507139705)

[6.1 Flow Chart of the Measurement Procedure 5](#_Toc507139706)

[6.2 Step by Step Description of the Measurement Procedure 5](#_Toc507139707)

[6.3 Definition and Equation of the Measurand 9](#_Toc507139708)

[6.4 Statistical Data Evaluation 9](#_Toc507139709)

[6.5 Reporting of the Results 9](#_Toc507139710)

[7 Potential Pitfalls 9](#_Toc507139711)

[8 Quality Control and Acceptance Criteria 9](#_Toc507139712)

[9 Health and Safety Warnings, Cautions and Waste Treatment 10](#_Toc507139713)

[10 Abbreviations 10](#_Toc507139714)

[11 References 10](#_Toc507139715)

[12 Annex 10](#_Toc507139716)

# Introduction

Inflammasomes are large intracellular multiprotein complexes that can respond to exogenous and endogenous danger signals and control the activation of caspase-1 (Martinon et al., 2002). Several types of inflammasomes are identified, each one having its unique activators and proteins that constitute the inflammasome. These proteins include the nucleotide-binding oligomerization domain (NOD), leucine rich repeat (LRR)-containing family members: Nod-like receptor family pyrin domain-containing 1 (NLRP1), NLRP3, NLRC4, absent in melanoma 2 (AIM2) and pyrin (Broz & Dixit, 2016).

The NLRP3 inflammasome is the best characterized inflammasome; it contains four main components: NLRP3, Nek7 kinase, pro-caspase-1 and ASC (apoptosis-associated speck-like protein containing CARD). ASC is an adaptor protein that bridges interactions between the other proteins (Schmid-Burgk et al., 2016). Several stimuli can activate the inflammasome, including pathogen-associated molecular patterns (PAMPs) of bacterial, viral, fungal and yeast origins, damage-associated molecular patterns (DAMPs) such as ATP, metabolic products and xenobiotics (Schroder & Tschopp, 2010) including nanomaterials (Sun et al., 2013). Upon activation, Nek7 kinase is recruited to the NLRP3 complex that binds pro-caspase-1 through ASC (see Figure 1) (Rathinam & Fitzgerald, 2016; Schmid-Burgk et al., 2016). Subsequently, caspase-1 is activated, which results in activation of the pro-inflammatory cytokines interleukin (IL)-1β and IL-18. Additionally, activated caspase-1 cleaves gasdermin D, which results in pyroptosis (Figure 1). Pyroptosis is an inflammatory type of programmed cell death that is characterized by swelling and membrane rupture (Broz & Dixit, 2016). **The assay for inflammasome activation described in this SOP is based on concomitant measurement of cell viability (viz. pyroptosis) and IL-β and IL-18 production.**

Sustained NLRP3 inflammasome activation may result in chronic inflammation and subsequent tissue damage. This sustained activation is implicated in a range of chronic diseases such obesity and type 2 diabetes (reviewed by Lukens et al. (2011) and De Nardo et al. (2014)) and Alzheimer’s disease (Venegas et al., 2017).


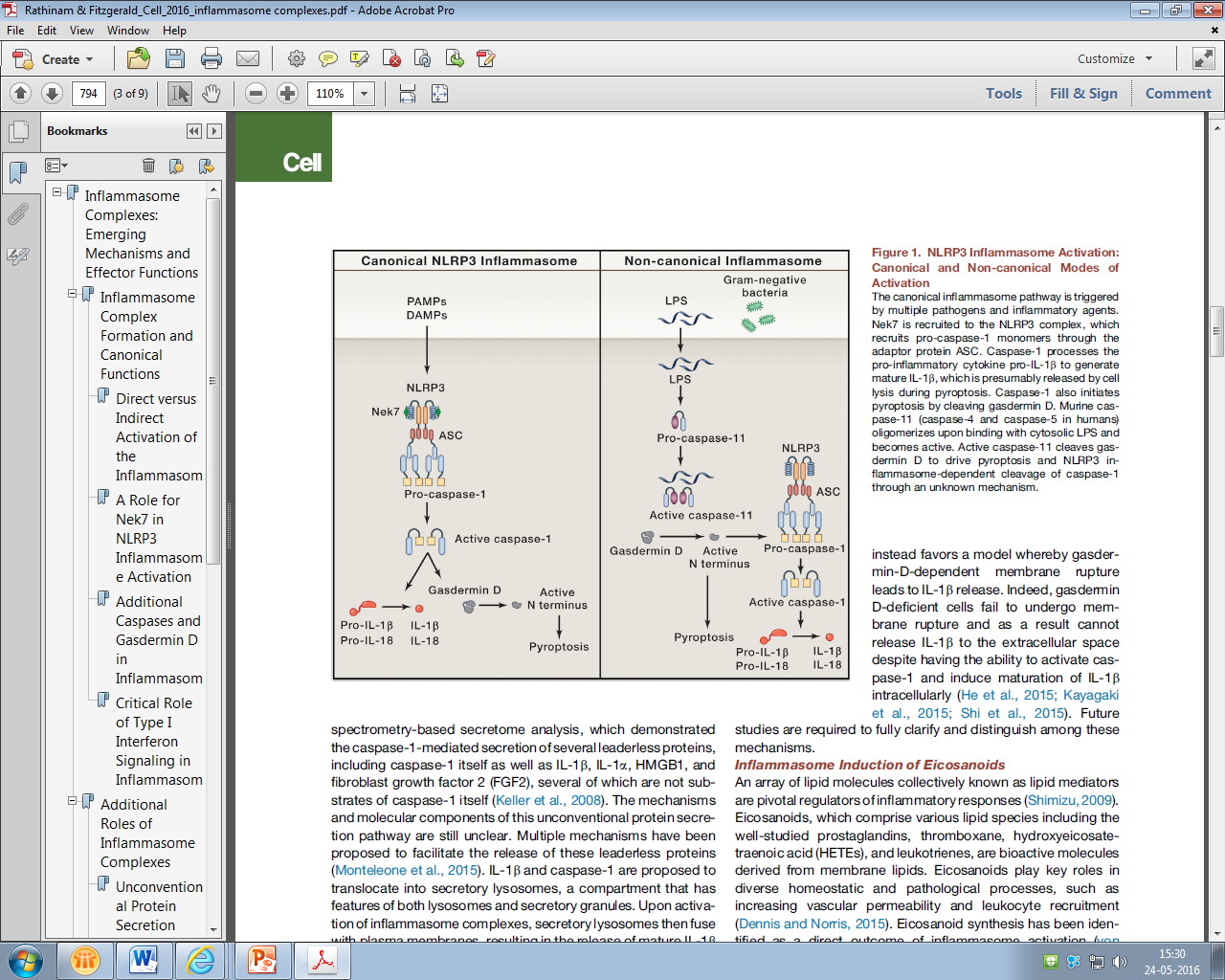


**Figure 1. Activation of the NLRP3 inflammasome.** Upon activation, NEK7 kinase is recruited to the NLRP3 complex, which binds pro-caspase-1 through the ASC adaptor protein. Subsequently, caspase-1 is activated, which results in activation of IL-1β and IL-18 and cleavage of gasdermin D and therefore pyroptosis.

# Principle of the Method

The principle of the evaluation of inflammasome activation is the measurement of the concomitant dose-dependent loss of viability and increase in IL-1β and IL-18 production.

# Applicability and Limitations (Scope)

Prior to deciding for which concentration range the NLRP3 inflammasome activation assay should be conducted, two issues need to be settled first. Firstly, is information available on (plasma) levels of the NMP? If so, a 10- or 100-times higher concentration should be used as the highest test concentration. If not, 100 µg/ml should be used as the highest test concentration. Secondly, the cytotoxicity of the NMP in the concentration range established should be tested. The concentration range should at least comprise two logs (I.e. 100-fold). Generally, in a stepwise approach of testing the NMP these deliberations have been made at an earlier stage.

The NMP should be tested for its cytotoxicity according to standard procedures, i.e. a well-dispersed NMP, sufficient incubation time (24 –48 hours), routinely used cell lines of human origin, e.g. A549 and THP-1, and a generally accepted way to evaluate viability, e.g. LDH release (membrane leakage) and WST-1 (mitochondrial function). Be aware of possible interference of the NMP in colorimetric determinations (as e.g. seen with Venofer). Select the concentration range where the effects on viability are below 20% (so, viability > 80% for both LDH release and WST-1). Please refer to dedicated SOPS.

In case a toxic compound in the NMP prohibits finding an appropriate dose range, the NLRP3 inflammasome activation assay should not be used as the results cannot be interpreted.

# Related Documents

Table 1:

| **Document ID** | **Document Title** |
| --- | --- |
| xxx | *Preparation and characterization of NMP dispersion* |
| xxx | *General cytotoxicity of NMP* |

# Equipment and Reagents

## Equipment

1. Centrifuge, e.g. Eppendorf Centrifuge 5810 R
2. Incubator with 5% CO_2_ and humidified atmosphere, e.g. Binder CB 150
3. ELISA plate washer e.g. BioTek 405 TS
4. Freezer (-20°C)
5. Freezer (-80°C)
6. Fridge (4°C)
7. Laminar flow cabinet, e.g. Clean Air EN 12469
8. Microscope, e.g. Nikon Labophot
9. Reversing microscope, e.g. Nikon TMS
10. Sonication bath, e.g. Branson Bransonic Ultrasonic Bath and/or a Vortex
11. Spectrophotometer, e.g. Molecular Devices SpectraMax M2
12. Water bath, e.g. Grant GD100
13. Cell culture Flask 75 cm2, e.g. Cellstar/Greiner Bio-One (#658175)
14. 96 wells tissue culture plate, flat bottom, e.g. Cellstar/Greiner Bio-One (#655180)

## Reagents

1. Foetal Calf Serum – Integro (Fischer Scientific, ThermoFischer) or Greiner Bio-One or Gibco (Thermo Scientific)
2. Hygromycin B Gold (CAS Number 31282-04-9) - InvivoGen ant-hg-1 (or ant-hg-5), 100 mg/ml
3. Human IL-1β ELISA kit, e.g. Invitrogen 88-7261-88 (ThermoFischer)
4. Human IL-18 ELISA kit, e.g. Invitrogen BMS267-2MST (ThermoFischer)
5. Nigericin (CAS Number 28643-80-3) - InvivoGen tlrl-nig (10 mg).
6. Penicillin/Streptomycin (10,000 U/mL) – Gibco (ThermoFischer)
7. PMA (phorbol 12-myristate 13-acetate; CAS Number 16561-29-8) - Sigma P8139-1MG or P8139-5MG
8. RPMI 1640, GlutaMAX™, HEPES - ThermoFischer 72400
9. THP-1 cells – ATCC TIB 202 (or THP-1null cells - InvivoGen)
10. THP1-def ASC cells – InvivoGen
11. THP1-def NLRP3 cells – InvivoGen
12. Water, ACS reagent, for ultratrace analysis - Sigma 14211-1L-F
13. PBS pH 7.2 (without CaCl2, MgCl2) – Gibco – #20012-019
14. WST-1 (CAS Number 150849-52-8) - Roche 05 015 944 001 (8 ml) or 11 644 807 001 (25 ml)

## Reagent Preparation

Before preparing cell culture medium, Foetal Calf Serum (FCS) should be de-complemented (i.e. complement inactivated), only if the serum has not yet decomplemented by the manufacturer :

5.3.1 Set the water bath temperature at 56°C, put the bottle in water the bath, wait about 30 min and check its temperature. Continuously gently shaking in the bath is preferable, in case the bath has this option.

5.3.2 When the temperature is 56°C, incubate the bottle for 30 min in the water bath.

5.3.3 In the flow cabinet, aliquot the de-complemented FCS in 50 ml portions and store at -20°C until use.

Preparation of complete cell culture medium:

5.3.4 Warm a bottle of 500 ml RPMI and a tube of 50 ml de-complemented FCS in a 37°C water bath for approximately 30 min.

5.3.5 Switch on the flow cabinet and sterilize the interior using 70% ethanol.

5.3.6 When RPMI and FCS have been warmed up, dry them with a disposable paper towel, towel, surface-sterilize them using 70% alcohol and move them to the flow cabinet. From now on, work in the flow cabinet.

5.3.7 Pipette the FCS up and down. Take the content of the tube (50 ml) and add it to the flask containing RPMI (500 ml).

5.3.8 Add 5 ml penicillin/streptomycin (pen/strep) to RPMI + FCS.

5.3.9 Close the lid of the bottle (containing RPMI + FCS + pen/strep) and invert the bottle several times.

5.3.10 Label the flask (cell type, operator’s name, date, ingredients).

NOTE: Complete cell culture medium cannot be used for more than 3 months.

# Procedure

## Flow Chart of the Measurement Procedure

| THP-1 cell culture  Differentiation of THP-1 cells to macrophages  Exposure of macrophages to NMP  Evaluation of cell viability and IL-1β production  If inflammasome activation is indicated, repeat experiment with ASC- and NLRP3-deficient THP-1 cells | Figure 2: Brief outline of the workflow. |
| --- | --- |

## Step by Step Description of the Measurement Procedure

Cell counting:

6.2.1 Visually check the cell culture medium and check the cell culture under the reversing microscope. Figure 3 provides an example what the cells should look like.


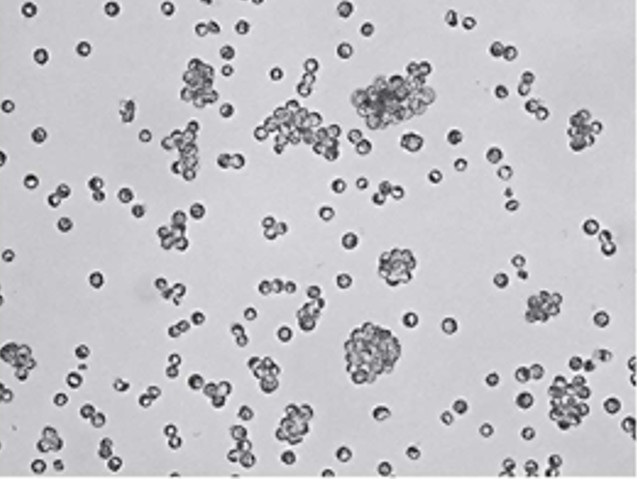


**Figure 3.** THP-1 monocytes.

6.2.2 In the flow cabinet, transfer the cell suspension to a 50 ml centrifuge tube.

6.2.3 Centrifuge at 130 g for 5 min (24°C; the setting of “accelerate” should be 8 (on a scale of 0-10); the setting of “brake” should also be 8 on a scale from 0-10).

6.2.4 During centrifugation, clean a “Burker” cell counting chamber using 70% ethanol and add 30 µl trypan blue to an unused well of a 96-wells plate.

6.2.5 After centrifugation, take the tube to the flow cabinet and check whether a cell pellet has formed. Pour off the supernatant. Add 2 ml complete cell culture medium (in case the cells come from one T75 Flask) to the pellet and re-suspend. Transfer 30 µl cell suspension to the 30 µl trypan blue already in the well. This way the cell suspension is diluted 2 times.

6.2.6 Pipette the cell culture/trypan blue mix, up and down and put it in the cell counting chamber.

6.2.7 Count the number of cells in 25 squares using 20x magnification. If there are too many cells to be reliably counted, make a new sample with another dilution factor. Remember to make the appropriate correction in the calculation of the cell concentration. An alternative option is to count only 4 or 12 squares and divide the cell number by 4 or 12 and multiply by 25. The formula for the concentration is based on the counting in 25 squares.

Concentration of the cells (cells/ml) = count number*dilution factor*10^4^ cells/ml

NOTE: The dilution factor = 2, unless another dilution is made.

6.2.8 For exposure experiments, prepare a cell concentration of 5x10^5^ cells/ml. Calculate the volume of cell suspension required for the experiment. First add the required volume of complete cell culture medium into a 50 ml tube and then add the required volume from the cell suspension. Mix by pipetting up and down.

6.2.9 For keeping the cells in culture, prepare a cell concentration of 2x10^5^ cells/ml. The total volume per flask is 15 ml. First add the required volume of complete cell culture medium into a new flask and then add the cells out of the suspension. Mix gently by pipetting up and down. Label the flask with cell type, operator’s name, date, and passage number. Passage is twice a week. This is critical. The scheme is Monday-Thursday or Tuesday-Friday. The cell concentration may not reach 1x10^6^/ml (in that case they start to differentiate). So, each time after collecting the cells, count backwards what the concentration was in the flask that you used.

NOTE THP-1 cells should not be passaged more than 20 times, starting with frozen cells with a low passage number. Keep them in culture maximal 3 months. Therefore, it is critical to prepare an adequate number of frozen stocks at early passages.

Additional antibiotics when using ASC-deficient and NLRP-3 deficient THP-1 cells.

6.2.10. Both cell types were generated using gene knockdown (Huang et al., 2009). Genetic instability is a biological phenomenon that occurs in all stably transfected cells. Therefore: at every other passage, THP1-def ASC cells and THP1-def NLRP3 cells should be maintained in complete cell culture medium supplemented with Hygromycin B Gold (200 µg/ml), so RMPI + FCS + pen/strep + hg (dilute the 100 mg/ml stock 500x).

Differentiation of THP-1 cells to macrophages:

6.2.11 In the flow cabinet, prepare the PMA stock solution by adding pure (96%) ethanol to the tube containing the PMA, until a final stock concentration of 100 µg/ml. Dissolve by inverting the tube several times and visually check for complete dissolution. Store aliquoted at -20°C. The solution will not freeze because of the ethanol. When using a vial, keep it closed as much as possible, to prevent evaporating the ethanol. Keep it cold when you take it out of the freezer, pipette quickly your required volume, and put back to the freezer immediately.

6.2.12 In the flow cabinet, take the 5x10^5^ cells/ml THP-1 cell culture (see above) and add by pipetting the appropriate volume of PMA stock solution such that a 1000-fold dilution of PMA is obtained. For instance, if the THP-1 cell culture has a volume of 5 ml, add 5 µl PMA stock solution. Mix the PMA and the cells by pipetting. Immediately put the PMA stock solution back at -20°C. The final PMA concentration with the cells is 100 ng/ml.

6.2.13 Add 100 µl of the cell suspension to each well of a 96-well flat bottom well plate, according to the layout depicted in Figure 4 (see next page). Preferably, use a multichannel pipette.

6.2.14 Label the plate with cell type, operator’s name, identity of NMP(s), date, and WST or ELISA.

6.2.15 Package the plate(s) with transparent film to prevent evaporation of medium. Put them in the incubator (37 °C, 5% CO_2_, humidified atmosphere) for 3 hours.

6.2.16 Take the plate(s) from the incubator. Check the confluence of the cells under the reversing microscope. The cells should now be attached the bottom of the wells. See Figure 5 below.


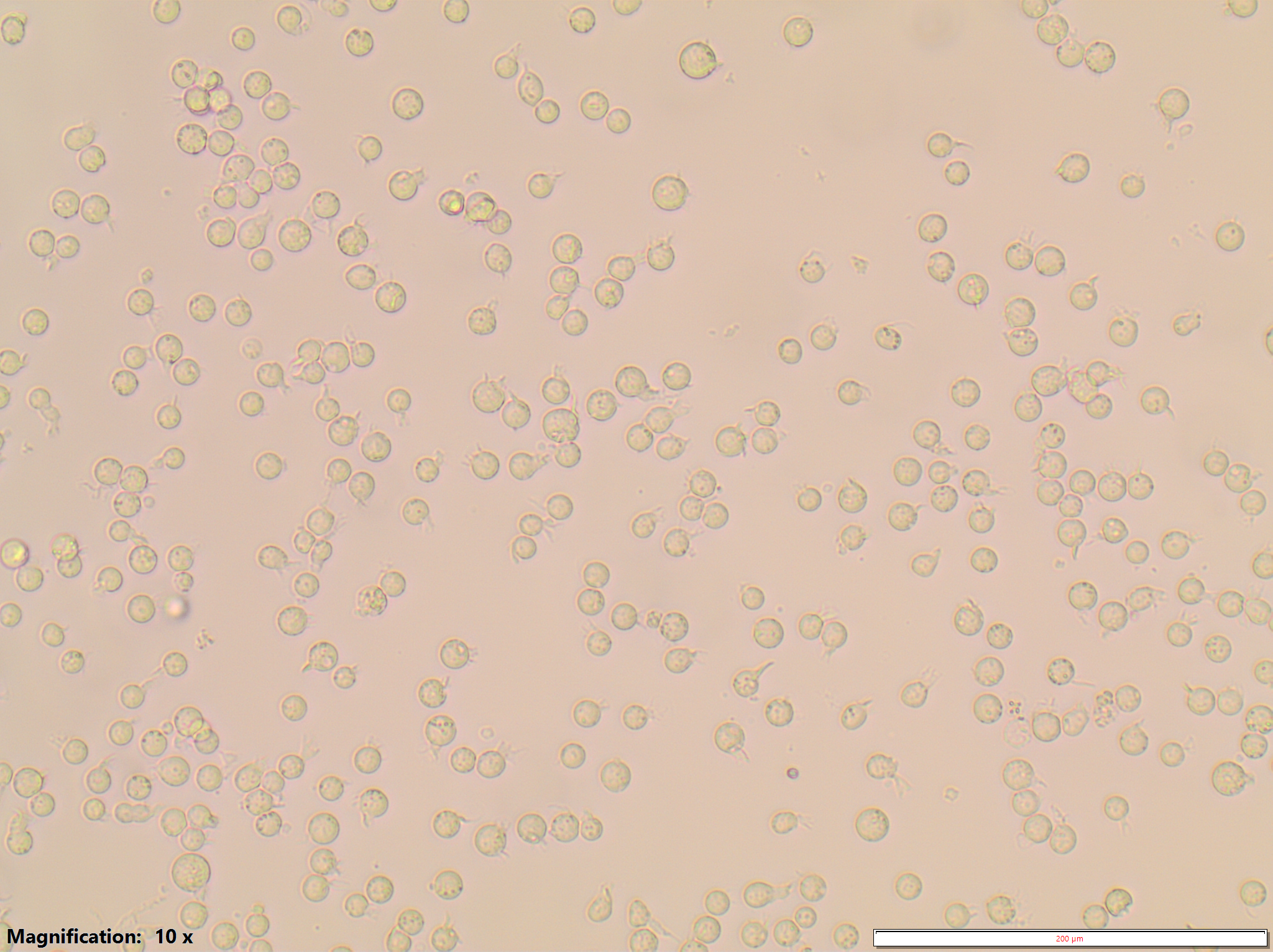


**Figure 5.** PMA-stimulated THP-1 cells.

6.2.17 Remove the medium from the wells. The pipette should be set at a higher volume than 100 µl to make sure all medium is removed. Tilt the plate a little bit and remove the medium from the corner each well. Preferably, use a multi-channel pipette, very carefully and slowly pipetting to not disturb the cell layer.

6.2.18 Add 100 µl fresh complete cell culture medium to each well (without PMA!).

6.2.19 Check the cells under the reversing microscope. See Figure 5. Package the plate(s) with transparent film and put them back in the incubator for 20-24 hours.

NP (µg/ml) nigericin (µg/ml)

0.625 1.25

0 2 4 8 16 32 64 128

[
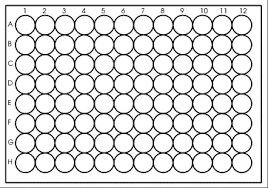
](https://www.google.com/url?sa=i&url=https%3A%2F%2Fopenaccess.leidenuniv.nl%2Fbitstream%2Fhandle%2F1887%2F22550%2FPROEF.KUANYAN.THESIS.pdf%3Fsequence%3D18&psig=AOvVaw3mI_qGuuKBT81CTFGMDsmR&ust=1587815449810000&source=images&cd=vfe&ved=0CAIQjRxqGAoTCLjT6tD_gOkCFQAAAAAdAAAAABDDAQ)Scheme for WST-1

without cells

with cells

NP (µg/ml) nigericin (µg/ml)

0.625 1.25

0 2 4 8 16 32 64 128

[
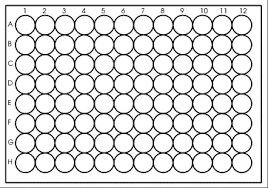
](https://www.google.com/url?sa=i&url=https%3A%2F%2Fopenaccess.leidenuniv.nl%2Fbitstream%2Fhandle%2F1887%2F22550%2FPROEF.KUANYAN.THESIS.pdf%3Fsequence%3D18&psig=AOvVaw3mI_qGuuKBT81CTFGMDsmR&ust=1587815449810000&source=images&cd=vfe&ved=0CAIQjRxqGAoTCLjT6tD_gOkCFQAAAAAdAAAAABDDAQ)Scheme for ELISA

with cells

**Figure 4.** Plate layout for WST-1 and ELISA measurement.

Exposure to NMP:

6.2.20 Prepare an NMP dispersion of 1.28 mg/ml (or other concentration). Previous work should have resulted in sufficient characterization of a similarly prepared dispersion. See SOP xxx.

6.2.21 Before preparing the concentration series of NMP(s), check the cells under the reversing microscope. See Figure 3.

6.2.22 In the flow cabinet, prepare the 2-fold dilution series of NMPs as follows (see next page). NOTE 1: the scheme depicted below takes 1.28 mg/ml NMP as a suspension to start with; of course, any other concentration can be taken as start. NOTE 2: In general, concentrations in the order of 100 µg/ml are the highest concentrations tested in *in vitro* studies. The example on the next page uses 128 µg/ml as highest concentration.

First, calculate the volume needed for each concentration (in this case, each dose requires exact 900 µl. Make a little more, e.g. 1100 µl).

a) Label 7 tubes for dispersion medium, which can be Ultrapure water, complete medium or in this case we used PBS (U) and 8 tubes for complete cell culture medium (M).

b) Add 300 µl PBS to each tube marked U and 880 µl medium to each tube marked M.

c) Sonicate the NMPs in a sonication bath at room temperature for 5 min if the NMPs require this, or vortex them 1-2 minutes. In this case (PACAs) we vortexed them.

d) Take 300 µl from the NMP suspension (of 1.28 mg/ml) and add it to a tube containing 300 µl PBS (U) to obtain a 0.64 mg/ml suspension. Mix by pipetting up and down. Then take 300 µl from the 0.64 mg /ml tube and add it to a tube containing 300 µl ultrapure water (U) to obtain a 0.32 mg/ml suspension. Proceed until a 0.02 mg/ml suspension is obtained. It is not necessary to change the tip of the pipette.

e) Take 220 µl from each U tube and add it to the corresponding M tube. Mix by pipetting up and down. Change the tip of the pipette every time. For the 128 µg/ml dispersion, take 220 µl directly from the 1.28 mg/ml stock and add it to 880 µl medium.

f) Check the total volume of each tube.

6.2.23 Remove carefully and slowly the medium from the wells with a multichannel pipet. Do not disturb the cell layer. Add carefully 100 µl fresh warm complete medium. Add 100 µl of each concentration NMP in medium to the corresponding wells. Pipette up and down before adding to the wells.

6.2.24 Package the plate with transparent film and put it in the incubator for 48 hours.

Exposure to the positive control Nigericin

a) Make a 5 mg/ml (= 6.7 mM) stock solution of Nigericin in 100% Ethanol

b) Aliquot this and store in the -20˚C

c) Take 2 concentrations of Nigericin to add to the cells : 0.625 and 1.25 µM as final concentration in the wells. Start with diluting the stock solution to 12.5 µM in PBS.

d) From this point, the procedure is the same as described for the NMP.

e) Figure 6 below shows what the positive control should look like.


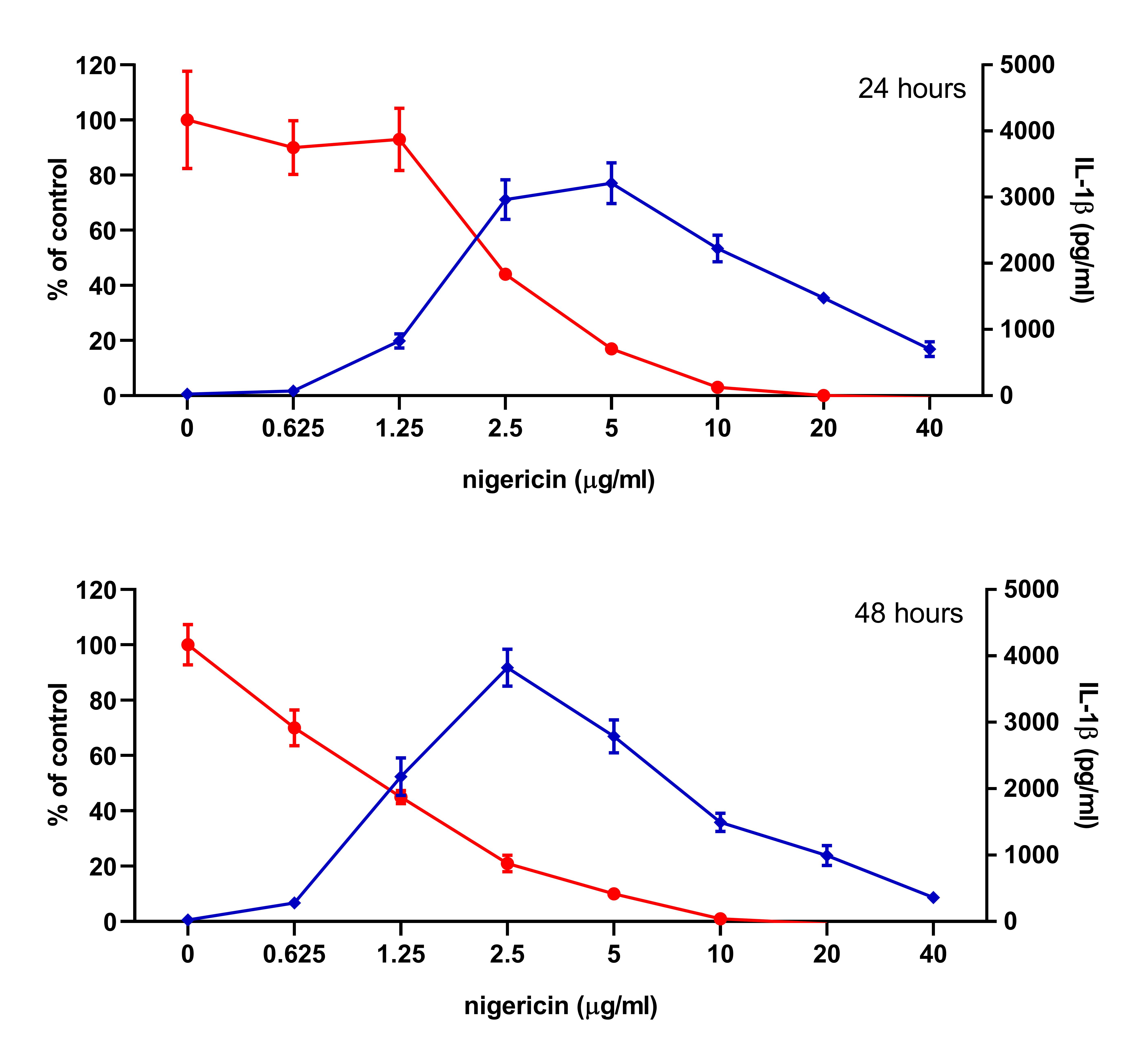


**Figure 6.** Viability (red; left axis) and IL-1β production (blue; right axis). Induction by a concentration range of the positive control nigericin.

**300 µl 300 µl 300 µl 300 µl 300 µl 300 µl**

**220 µl 220 µl 220 µl 220 µl 220 µl 220 µl 220µl 220 µl**

**Figure 7.** Dilution scheme of NMP.

Cell viability measurement:

6.2.25 Check the cells under the reversing microscope. The cells that were not exposed to the NMP should look like Figure 3.

6.2.26 Take WST-1 from -20°C and thaw.

6.2.27 Put WST-1 and the cell culture plate in the flow cabinet. Add 20 µl WST-1 to each well. Preferably use a multi-channel pipette. Do not touch the medium with the pipette tips; it is not necessary to change the pipette tips. Mix on the table by performing an 8-drawing.

6.2.28 Put the plates in the incubator. Incubate the cells for 1, 2 and 3 hours. Measure the absorbance with a spectrometer at 440 nm (420-480 nm advised) versus a 620 nm reference. The absorbance should be 1.5-3.0; if this is not reached after 3 hours, measure again after 4 hours (so 1 hour later).

6.2.29 Calculate the cell viability:

1. For each NMP concentration (including control without NMP) calculate the average absorbance value of sample with cells (denoted X) (N=4 test samples; blue square in Figure 4) and that of sample without cells (denoted Y) (N=2 interference samples; red square).
2. For each NMP concentration (denoted C), subtract the absorbance of the interference samples from those of the test samples (so, X-Y for each C). This should also be done for the control without NMP (denoted C0).
3. Cell Viability = (X-Y (C) / X-Y (C0)) x 100%.

IL-1β ELISA

It is advised to purchase ELISA kits, not antibody pairs. It is not necessary to purchase kits with coated plates, or kits with plates included. The ELISA can be performed according to the manufacturer’s instructions. Nunc Maxisorp plates perform well for a wide range of ELISA’s.

6.2.30 Importantly, for IL-1β ELISA on PMA-stimulated THP-1 cells, for the measurements to be within the range of the calibration curve, the samples should be diluted in assay diluent from the Elisa Kit, 10 or 25 times before measurement.

IL-18 ELISA

It is advised to purchase ELISA kits, not antibody pairs. It is not necessary to purchase kits with coated plates, or kits with plates included. The ELISA can be performed according to the manufacturer’s instructions. Nunc Maxisorp plates perform well for a wide range of ELISA’s.

IL-18 can be measured to confirm the results on IL-1β. Literature either reports IL-1β results only or results on both IL-1β and IL-18. Therefore, we first perform an IL-1β ELISA. Only if confirmation of the results is required, e.g. when setting up the assay, an IL-18 ELISA is performed.

6.2.31 In our experience, although the concentrations of IL-18 are much lower compared to IL-1β the dose-response curves are similar. For IL-18 ELISA on PMA-stimulated THP-1 cells, it is not necessary to dilute the samples before measurement.

## Definition and Equation of the Measurand

Two sets of data are generated: viability as a function of NMP concentration, and IL-1β concentration as a function of NMP concentration. Only in rare occasions, IL-18 concentration as a function of NMP concentration is generated as well.

## Statistical Data Evaluation

There is no generally accepted method for statistical analysis of NLRP3 inflammasome activation. The following analyses may be performed: Part A provides information whether NLRP3 inflammasome activation has indeed taken place, while part B provides information on the potency of the NMP to activate the NLRP3 inflammasome.

1. Basic statistical analysis.

6.4.1 Perform dose-response analysis on viability and IL-1β (and IL-18) production. A statistically significant decrease in viability and increase in IL-1β (and IL-18) production indicates inflammasome activation.

6.4.2 Perform the same analysis using THP1-def ASC cells and THP1-def NLRP3-def cells. A statistically significant decrease in viability and in IL-1β (and IL-18) production of the two deficient cell lines compared to the wildtype cells indicates inflammasome activation.

B. Dose-response modelling.

6.4.3. Perform dose response modelling on both viability and IL-1β (and IL-18) production, using the benchmark dose approach with PROAST software (Slob, 2002) ([www.rivm.nl/proast](http://www.rivm.nl/proast)).

In this way the concentration that results in 50% reduction in viability calculated (EC50 value plus 90% confidence interval). Also, the concentration that results in 50% increase in IL-1β (and IL-18) production is calculated (EC50 value plus 90% confidence interval). In contrast to basic statistical analysis, this way of dose-response modelling makes use of all data points.

## Reporting of the Results

Basic statistical analysis:

Graphical representation of the results on THP-1 cells showing both viability and IL-1β production vs. the NMP concentration, including concentrations where statistical differences are seen compared to the control that contains no NMP. Similar graphical representations for THP1-def ASC cells and THP1-def NLRP3-def cells, including concentrations where statistical differences are seen compared to the THP-1 cells exposed to the same NMP concentration.

Benchmark dose modelling:

Concentration that gives 50% loss of viability (including its 90% confidence interval). Concentration that gives 50% induction of IL-1β (and IL-18) production (including its 90% confidence interval).

# Potential Pitfalls

Since LPS induces the production of pro-IL-1β and pro-IL-18, LPS may possibly affect the presence of IL-1β and IL-18. Infections of the cell culture medium may activate the NLRP3 inflammasome.

# Quality Control and Acceptance Criteria

THP-1 cells:

- The positive control nigericin should give a concentration-dependent decrease in the viability and at the same time a concentration-dependent increase in IL-1β production.
- At the next higher NMP concentration, viability should be similar or decreased. At the same time, at the next higher NMP concentration, IL-1β production should be similar or increased.

THP1-def ASC cells and THP1-def NLRP3-def cells:

- Viability and IL-1β production should not be affected by the NMP.
- IL-1β production should be negligible. NOTE: at higher NMP concentrations NLRP3-deficient cells give a somewhat higher, but still very low, IL-1β production than ASC-deficient cells.

# Health and Safety Warnings, Cautions and Waste Treatment

We consider all nanomaterials, including NMPs, as carcinogenic. Weighing of nanomaterials should therefore be done in a fume hood. Never open a vial of nanomaterials powder outside a fume hood or flow cabinet. Waste should be disposed of as carcinogenic waste. Always wear gloves during handling.

# Abbreviations

- ASC, apoptosis-associated speck-like protein containing CARD
- FCS, foetal calf serum
- NLRP3, Nod-like receptor family pyrin domain-containing 3
- NMP, nanomedicinal product

# References

Broz P, Dixit VM. Inflammasomes: mechanism of assembly, regulation and signalling. Nat Rev Immunol. 2016;16(7):407-20.

De Nardo D, De Nardo CM, Latz E. New insights into mechanisms controlling the NLRP3 inflammasome and its role in lung disease. Am J Pathol. 2014;184(1):42-54.

Huang MT, Taxman DJ, Holley-Guthrie EA, Moore CB, Willingham SB, Madden V, Parsons RK, Featherstone GL, Arnold RR, O'Connor BP, Ting JP. Critical role of apoptotic speck protein containing a caspase recruitment domain (ASC) and NLRP3 in causing necrosis and ASC speck formation induced by Porphyromonas gingivalis in human cells. J Immunol. 2009;182(4):2395-404.

Lukens JR, Dixit VD, Kanneganti TD. Inflammasome activation in obesity-related inflammatory diseases and autoimmunity. Discov Med. 2011;12(62):65-74.

Martinon F, Burns K, Tschopp J. The inflammasome: a molecular platform triggering activation of inflammatory caspases and processing of proIL-1beta. Mol Cell. 2002;10(2):417-26.

Rathinam VA, Fitzgerald KA. Inflammasome complexes: emerging mechanisms and effector functions. Cell. 2016;165(4):792-800.

Schmid-Burgk JL, Chauhan D, Schmidt T, Ebert TS, Reinhardt J, Endl E, Hornung V. A Genome-wide CRISPR (Clustered Regularly Interspaced Short Palindromic Repeats) Screen Identifies NEK7 as an Essential Component of NLRP3 Inflammasome Activation. J Biol Chem. 2016;291(1):103-9.

Schroder K, Tschopp J. The inflammasomes. Cell. 2010;140(6):821-32.

Slob W. Dose-response modeling of continuous endpoints. Toxicol Sci. 2002;66(2):298-312.

Sun B, Wang X, Ji Z, Li R, Xia T. NLRP3 inflammasome activation induced by engineered nanomaterials. Small. 2013;9(9-10):1595-607.

Venegas C, Kumar S, Franklin BS, Dierkes T, Brinkschulte R, Tejera D, Vieira-Saecker A, Schwartz S, Santarelli F, Kummer MP, Griep A, Gelpi E, Beilharz M, Riedel D, Golenbock DT, Geyer M, Walter J, Latz E, Heneka MT. Microglia-derived ASC specks cross-seed amyloid-β in Alzheimer's disease. Nature. 2017;552(7685):355-61.

Yazdi AS, Guarda G, Riteau N, Drexler SK, Tardivel A, Couillin I, Tschopp J. Nanoparticles activate the NLR pyrin domain containing 3 (Nlrp3) inflammasome and cause pulmonary inflammation through release of IL-1alpha and IL-1beta. Proc Natl Acad Sci U S A. 2010;107(45):19449-54.

# Annex
