# Supplementary material for: An inter-laboratory comparison of an NLRP3 inflammasome activation assay and dendritic cell maturation assay using a nanostructured lipid carrier and a polymeric nanomedicine, as exemplars
Source: Drug Deliv Transl Res. 2022 Jul 15;12(9):2225–42. doi: 10.1007/s13346-022-01206-6 (PMC9360168; doi:10.1007/s13346-022-01206-6)
Supplement: Supplementary file 2 — Supplementary file2 (DOCX 211 KB) [file 13346_2022_1206_MOESM2_ESM.docx]

| Project: | 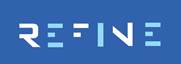 |
| --- | --- |
| Dendritic cell maturation by nanomedicinal products | |
| Subtitle | |

| AUTHORED BY: | DATE: |
| --- | --- |
| Rob Vandebriel, PhD | February 27, 2019 |

| REVIEWED BY: | DATE: |
| --- | --- |
|  |  |
|  |  |

| APPROVED BY: | DATE: |
| --- | --- |
|  | DD/MM/YYYY |

DOCUMENT HISTORY

| Effective Date | Date Revision Required | Supersedes |
| --- | --- | --- |
| DD/MM/YYYY | DD/MM/YYYY | DD/MM/YYYY |

| Version | Approval Date | Description of the Change | | Author / Changed by |
| --- | --- | --- | --- | --- |
| 1.0 | DD/MM/YYYY | All | Initial Document | Name |

Table of Content

[1 Introduction 3](#_Toc507139697)

[2 Principle of the Method 3](#_Toc507139698)

[3 Applicability and Limitations (Scope) 3](#_Toc507139699)

[4 Related Documents 4](#_Toc507139700)

[5 Equipment and Reagents 4](#_Toc507139701)

[5.1 Equipment 4](#_Toc507139702)

[5.2 Reagents 4](#_Toc507139703)

[5.3 Reagent Preparation 5](#_Toc507139704)

[6 Procedure 7](#_Toc507139705)

[6.1 Flow Chart of the Measurement Procedure 7](#_Toc507139706)

[6.2 Step by Step Description of the Measurement Procedure 7](#_Toc507139707)

[6.3 Definition and Equation of the Measurand 16](#_Toc507139708)

[6.4 Statistical Data Evaluation 16](#_Toc507139709)

[6.5 Reporting of the Results 16](#_Toc507139710)

[7 Potential Pitfalls 16](#_Toc507139711)

[8 Quality Control and Acceptance Criteria 16](#_Toc507139712)

[9 Health and Safety Warnings, Cautions and Waste Treatment 16](#_Toc507139713)

[10 Abbreviations 16](#_Toc507139714)

[11 References 17](#_Toc507139715)

[12 Annex 17](#_Toc507139716)

# Introduction

The assay described here measures the capacity of a NMP to (inadvertently) stimulate the adaptive immune response. The assays measures maturation of DC based on effects on DC surface marker expression and cytokine production by DC. Many nanoparticles and some NMP have been shown to exert this activity, which is in principle adverse.

# Principle of the Method

(1) Buffy coats are isolated from blood that is obtained from human donors, (2) monocytes are purified from these buffy coats, (3) these purified monocytes are differentiated to immature DC, (4) these immature DC are exposed to NMP, and (5) the maturation status of DC is measured by cell surface marker expression and cytokine production.

Dendritic cells (DC) are sentinel cells that are pivotal in the initiation of adaptive immune responses (Banchereau & Steinman, 1998). Moreover, they integrate various stimuli, such as from different microbe associated molecular patterns (MAMPs) and the cytokine milieu. MAMPs are detected by pattern recognition receptors (PRRs) that are highly expressed by DC. PRRs can be located on the cell surface, in endocytic compartments, or in the cytoplasm. Important classes of PRRs are the Toll-like receptors (TLRs) and Nod-like receptors (NLRs). The nature of the immune response following DC function is greatly influenced by the PRR (or combination of different PRRs). In this way, DC form an important link between the innate and adaptive immune response.

DC appear as immature DC which are very well capable of ingesting protein antigens and as mature DC which are especially capable of presenting peptides to naive T-cells. This process of DC maturation is central to the functioning of DC. DC maturation can be readily measured based on cell surface marker expression, mostly using FACS, and cytokine production, mostly using ELISA-based assays. Nanomedicinal products (NMP) are able to influence DC maturation and by that immune function. For example, DC-SIGN-targeting galactofuranose-functionalized 2 nm Au NP induce DC maturation (Chiodo et al., 2014). PEGylated 12 nm Au NP are another example of NMP that induce DC maturation (Fernández et al., 2015).

For *in vitro* studies, DC are either isolated from murine bone marrow (bone marrow DC or BMDC) or derived from human peripheral blood monocytes (monocyte-derived DC or MDDC, also named moDC). MoDC are a popular cell type to study DC function *in vitro*, for various reasons: (1) since they can be cultured from monocytes isolated from human blood, they are readily available in large numbers, (2) no cross-species extrapolation is required, and (3) no laboratory animals are required. It should be noted, however, that the *in vivo* equivalent of moDC has been difficult to identify (Satpathy et al., 2012).

The panel of cell surface markers used to measure moDC maturation generally comprises HLA-DR (MHC class II) and the T-cell co-receptors CD40, CD80, and CD86, although additions to this panel (e.g. CD54, CD83, CD206* (mannose receptor), CD209* (DC-SIGN), HLA-A/B/C, CCR7, and PD-L1) are sometimes included while in other studies the panel used is more limited. The cytokines measured to evaluate DC maturation are mostly IL-12p40 or IL-12p70, but also IL-10 and TNF-α.

*CD206 and CD209 may be used as markers for iDC.

# Applicability and Limitations (Scope)

Prior to deciding for which concentration range the DC maturation assay should be conducted, two issues need to be settled first. Firstly, is information available on (plasma) levels of the NMP? If so, a 10- or 100-times higher concentration should be used as the highest test concentration. If not, 100 µg/ml should be used as the highest test concentration. Secondly, the cytotoxicity of the NMP in the concentration range established should be tested. The concentration range should at least comprise two logs (I.e. 100-fold). Generally, in a stepwise approach of testing the NMP these deliberations have been made at an earlier stage.

The NMP should be tested for its cytotoxicity according to standard procedures, i.e. a well-dispersed NMP, sufficient incubation time (24 –48 hours), routinely used cell lines of human origin, e.g. A549 and THP-1, and a generally accepted way to evaluate viability, e.g. LDH release (membrane leakage) and WST-1 (mitochondrial function). Be aware of possible interference of the NMP in colorimetric determinations (as e.g. seen with Venofer). Select the concentration range where the effects on viability are below 20% (so, viability > 80% for both LDH release and WST-1). Please refer to dedicated SOPS.

In case a toxic compound in the NMP prohibits finding an appropriate dose range, the DC maturation assay should not be used as the results cannot be interpreted.

This assay uses monocyte-derived DC (moDC). As indicated above, an *in vivo* equivalent of moDC has not been identified yet.

To our knowledge, the DC maturation assay has been found to be applicable to all NMP tested so far (liposomes, SPIONs and a range of Au NP). However, care should be taken that the appropriate controls are met: (1) since LPS is able to induce DC maturation, care has to be taken that the NMP tested are not contaminated with LPS. LPS contamination may be checked by the Limulus amoebocyte lysate (LAL) assay or by a recently developed LC-MS/MS assay (REFINE SOP in progress; Giannakou et al. in press), (2) NMP; especially NMP containing an active pharmaceutical ingredient may be toxic to DC. Therefore, a cell viability assay (e.g. WST-1) should be included, (3) NMP may interfere with the optical read-outs that are inherent to both FACS and ELISA. In the FACS, this should be controlled for by including cells and NMP but without labelled antibodies, and in the ELISA by including NMP but without labelled antibodies, (4) mediators (such as cytokines) and substrates (such as WST-1) may possibly attach (in part) to NMPs. This is outside the scope of this SOP.

# Related Documents

Table 1:

| **Document ID** | **Document Title** |
| --- | --- |
| Xxx | *Preparation and characterization of NMP dispersion* |
| Xxx | *LC-MS/MS assay for endotoxin measurement (in progress)* |

# Equipment and Reagents

## Equipment

1. Centrifuge, e.g. Eppendorf Centrifuge 5810 R
2. Incubator with 5% CO_2_ and humidified atmosphere, e.g. Binder CB 150
3. ELISA plate washer e.g. BioTek 405 TS
4. FACS machine, e.g. FACS Canto II with FACS Diva software
5. Freezer (-20°C)
6. Freezer (-80°C)
7. Fridge (4°C)
8. Laminar flow cabinet, e.g. Clean Air EN 12469
9. Microscope, e.g. Nikon Labophot
10. Multistand, MACS Miltenyi Biotec
11. Reversing microscope, e.g. Nikon TMS
12. Shaker, e.g. GFL 3006 Analogue Reciprocating Shaker
13. Sonication bath, e.g. Branson Bransonic Ultrasonic Bath
14. Spectrophotometer, e.g. Molecular Devices SpectraMax M2
15. Water bath, e.g. Grant GD100

Note: Most of this equipment may be replaced by other brands or types, with similar specifications, whereas some equipment (i.e. the MACS product line) may only be purchased from the indicated manufacturer.

## Reagents

1. BSA Bovine Serum Albumin lyophilized powder - Sigma A9418
2. CD14 microbeads MACS, Miltenyi Biotec, 130-050-201
3. cell culture plate 12-well flat bottom e.g. Corning Costar 3513
4. cell culture plate 96-well V bottom e.g. BD Falcon 353263
5. CellFIX Becton Dickinson 340181
6. EDTA (CAS Number 6381-92-6) UltraPure 0.5M EDTA pH 8.0 - Invitrogen 15575-038
7. FCS HyClone Foetal Bovine Serum - Thermo Scientific SH30071.02. FCS should be de- complemented prior to use. See below.
8. Filter Nalgene 595-4520 rapid-flow filter
9. GM-CSF PeproTech 300-03B
10. IL-4 Active Bioscience 1515.951.025
11. KHCO_3_ (CAS Number 298-14-6) for analysis EMSURE® ACS - Merck 104854
12. Lymphoprep Axis Shield, Oslo, Norway Ficoll – 1114547
13. LS column MACS Miltenyi Biotech, 130-042-401
14. NH_4_Cl (CAS Number 12525-02-9) for analysis EMSURE® ACS, ISO, Reag. Ph Eur – Merck 101145
15. PBS Sterile PBS without CaCl_2_ and MgCl_2_, pH 7.2 - Gibco 20012-019
16. Pen/strep 10.000 U/ml Pen + 10.000 µg/ml Strep – Gibco 15140-122
17. RPMI RPMI 1640 glutamax - Gibco 72400
18. Trypan blue (CAS Number 72-57-1) e.g. ThermoFisher 15250061
19. Water (CAS Number 7732-18-5) ACS reagent, for ultratrace analysis - Sigma 14211-1L-F
20. WST-1 (CAS Number 150849-52-8) Roche 11 644 807 001 (25 ml)

Antibody panel for FACS: see Annex 1

Notes: Most of these reagents may be replaced by other brands or types, with similar specifications, while several reagents (i.e. CD14 microbeads, CellFIX, Lymphoprep) may only be purchased from the indicated manufacturer. **FCS, IL-4 and GM-CSF are all critical for proper differentiation of immature DC from monocytes. They should be tested for performance prior to NMP testing.**

The antibody panel shown in Annex 1 is currently used in RIVM, the Netherlands. If preferred, other panels may be used instead.

## Reagent Preparation

5.3.1 MACS buffer

5.3.1.1 PBS 500 ml

5.3.1.2 EDTA (2 mM final conc.) 2 ml of 0.5 M stock

5.3.1.3 BSA (0.5% m/v final conc.) 2.5 g

5.3.1.4 Add EDTA and BSA, to PBS. To let BSA dissolve, stir at RT for 15 min (or at 4°C overnight)

5.3.1.5 Before first use, filter sterilize using a 0.2 µm filter

5.3.1.6 Store at 4°C.

5.3.2 ACK buffer

5.3.2.1 Water (ACS reagent) 500 ml

5.3.2.2 NH_4_Cl (156 mM final conc.) 4.1722 g

5.3.2.3 KHCO_3_ (10 mM final conc.) 0.5006 g

5.3.2.4 EDTA (0.1 mM final conc.) 0.1 ml (of 0.5 M stock)

5.3.2.5 Leave to dissolve; set pH to 7.3

5.3.2.6 Before first use, filter sterilize using a 0.2 µm filter

5.3.2.7 Store at 4°C.

5.3.3 Cell culture medium

5.3.3.1 RPMI 8.9 ml

5.3.3.2 10% HyClone de-complemented FCS 1 ml

5.3.3.3 1% pen/strep 100 µl

5.3.3.4 450 U/ml GM-CSF 10 µl

5.3.3.5 500 U/ml IL-4 6.25 µl

Notes:

5.3.3.6 RPMI and FCS should be pre-heated in a 37°C water bath before preparing the medium. This is because especially IL-4 is unstable.

5.3.3.7 It should be stressed that the source of FCS, GM-CSF and IL-4 may profoundly affect the generation of immature DC.

5.3.3.8 Cell culture medium should be freshly made for each experiment.

5.3.4 Exposure medium

5.3.4.1 RPMI 8.9 ml

5.3.4.2 10% HyClone de-complemented FCS 1 ml

5.3.4.3 1% pen/strep 100 µl

5.3.4.4 450 U/ml* GM-CSF 11.54 µl

5.3.4.5 500 U/ml* IL-4 7.21 µl

*After adjustment to the final volume of 750 µl (see below)

Notes:

5.3.4.6 RPMI and FCS should be pre-heated in a 37°C water bath before preparing the medium. This is because especially IL-4 is unstable.

5.4.3.7 It should be stressed that the source of FCS, GM-CSF and IL-4 may profoundly affect the quality of the DC maturation assay.

5.3.4.8 Exposure medium should be freshly made for each experiment.

5.3.5 De-complementation of Foetal Calf Serum

Foetal Calf Serum (FCS) should be de-complemented (i.e. complement inactivated) before use:

5.3.5.1 Set the water bath temperature at 56°C, put in the FCS-containing bottle, wait about 30 min and check its temperature.

5.3.5.2 Incubate the bottle for 30 min in the water bath at 56°C.

5.3.5.3 Switch on the laminar flow cabinet and sterilize the interior using 70% ethanol.

5.3.5.4 Dry the bottle with a disposable paper towel, move it to the flow cabinet and surface-sterilize it using 70% ethanol. From now on, work in the flow cabinet.

5.3.5.5 Aliquot the de-complemented FCS in 50 ml portions and store at -20°C until use.

# Procedure

## Flow Chart of the Measurement Procedure

| Isolation of PBMC from buffy coat  Purification of monocytes from PBMCs  Differentiation of immature DC from monocytes  Exposure of immature DC to NMP  Measurement of cytokines by ELISA  Measurement of cell surface markers by FACS | Figure 1: Brief outline of the workflow.  Measurement of cytotoxicity |
| --- | --- |

## Step by Step Description of the Measurement Procedure

6.2.1 Isolation of PBMC from buffy coat using MACS beads

When needed prepare ACK and/or MACS buffer one day before isolation (see chapter 5 of this SOP). Use cold MACS buffer during the whole procedure. Use ACK buffer at room temperature.

6.2.1.1 In the laminar flow cabinet, put the buffy coat into two 50 ml tubes (each ≈ 25 ml).

6.2.1.2 In each tube, add PBS to 50 ml.

6.2.1.3 Divide the blood-PBS mixture in 4 new 50 ml tubes, 25 ml in each tube.

6.2.1.4 In each tube slowly lay ≈ 13 ml lymphoprep underneath the blood-PBS mixture, as follows: fill a 10 ml pipette with lymphoprep. Remove the filled pipette from the pipette boy and immediately plug the top of the pipette with the thumb to keep the lymphoprep from running out of the pipette. Place the pipette tip at the bottom of the tube and allow the lymphoprep to flow underneath the blood-PBS mixture. Make sure the first 4 ml are released from the pipette very slowly by putting a finger on top of the pipette. Leave 1 ml in the pipette. Plug the top of the pipette with the thumb when removing the pipette from the tube.

6.2.1.5 Centrifuge for 30 min at 1000*g, 25°C, “accelerate” = 5, “brake” = 0. Slow acceleration and brake is important.

6.2.1.6 In the flow cabinet, remove the upper layer containing plasma and most of the platelets. Use a 10 ml pipette.

6.2.1.7 The PBMC layer is a whitish turbid small fraction of 2-3 mm in thickness. Use a 10 ml pipette. Place the tip of the pipette in the middle of the PBMC layer and remove the PBMCs in one go. Avoid transferring erythrocytes.

6.2.1.8 Pool PBMC from two tubes in one 50 ml tube. Therefore, there are two tubes in total.

6.2.1.9 ill up the two tubes with PBS, each one to 50 ml.

6.2.1.10 Centrifuge for 8 min at 500 x g, 18-20°C; “brake” = 5.

6.2.1.11 Make sure ACK buffer is at room temperature. In the flow cabinet, remove the supernatant using a pipette, pool PBMC in one 50 ml tube (use ACK buffer to rinse the other tube). Add ACK buffer to 25 ml.

6.2.1.12 Centrifuge for 5 min at 500 x g, 18-20°C.

6.2.1.13 In the flow cabinet, remove the supernatant, re-suspend the cells in 50 ml cold MACS buffer and keep them on ice until centrifugation (step 16).

6.2.1.14 Add 20 μl of the cell suspension to 180 μl Trypan Blue (final dilution of 1:10) and count the cells.

6.2.1.15 Set aside two samples, each of 2x10e^5^ cells, for purity check. In case compensations have to be set on the FACS machine, set aside three additional samples, also each of 2x10e^5^ cells: for CD14-PE staining, live/dead-Aqua staining, and an unstained control.

6.2.1.16 Add cold FACS buffer in each well until a volume of 100 µl. Keep at 4°C until staining starts.

6.2.1.17 Centrifuge for 8 min at 500 x g, 5°C.

6.2.1.18 Decide how many PBMC you want to use for isolation of monocytes (isolation of more monocytes requires more beads, so do not isolate more monocytes than required for the experiment). Note: at least 8% of the PBMC are monocytes.

Total cell number (cells/ml x 50 ml) (cT): = ____________

Cell number for monocyte isolation (cM): = ____________

6.2.2 Monocyte isolation by CD14 positive selection

6.2.2.1 Remove the supernatant, re-suspend in MACS buffer (95 µl per 10^7^ cells):

Total volume: ____________ cells / 10^7^ * 95 µl = ____________ µl MACS buffer

6.2.2.2 Transfer cells for MACS isolation to a new 50 ml tube:

(cM / cT) * total volume = ____________ µl

6.2.2.3 Add 5 µl CD14 microbeads per 10^7^ cells of cM:

cM / 10^7^*5 µl= ____________ µl microbeads

6.2.2.4 Incubate for 15-30 min at 4°C while shaking (100/min).

6.2.2.5 Add MACS buffer to a final volume of 50 ml and centrifuge for 8 min at 500 x g, 4°C.

6.2.2.6 Insert the MACS column in the Multistand. Prepare a MACS column by washing it once with 3 ml MACS buffer. Throw away the flow-through.

6.2.2.7 Completely remove the supernatant and re-suspend the cell pellet in MACS buffer (500 µl per 10^8^ cells); avoid bubbles.

cM / 10^8^ * 500 µl = ____________ µl MACS buffer

6.2.2.8 Apply the cell suspension to the MACS column. After complete flow-through, rinse the column 3 times, using 3 ml MACS buffer each time. The combined flow-through is the CD14-negative cell fraction.

6.2.2.9 After complete flow-through, remove the MACS column from the Multistand and place the column over a clean tube. Add 5 ml MACS buffer to the column and quickly push the plunger through the column to obtain the CD14-positive cells. Keep the cells on ice.

6.2.2.10 Add 20 µl of the CD14-positive cells to 180 µl Trypan Blue (1:10) and count the cells.

Total CD14^+^ cells: (cells/ml x 5 ml) = ____________ CD14^+^ cells

CD14^+^ yield: CD14^+^ cells / cM *100= ____________ %

6.2.2.11 For a purity check, set a sample aside from the CD14-positive and the CD14-negative fraction. This sample should contain 2x10e^5^ cells. For the CD14-positive fraction, calculate the volume to get this number of cells. Use the same volume for the CD14-negative fraction.

6.2.2.12 Add MACS buffer until 100 µl per well.

6.2.3 Seeding of CD14^+^ monocytes and differentiation to immature DC

6.2.3.1 Take the number of cells needed (3.6x10^5^ cells per exposure) and spin down for 8 min at 500 x g, 4°C.

6.2.3.2 In the flow cabinet, re-suspend the cell pellet in cell culture medium to 3x10^5^ cells per ml. Seed in a 12-well plate 1 ml/well. Seed in a 96-well plate 200 µl/well. Note: the 12-well plate is for cell surface marker expression and cytokine production (FACS and ELISA, respectively); the 96-well plate is for viability measurement (WST-1).

6.2.3.3 Package the plates with transparent film to prevent evaporation of medium. Put them in the incubator (37°C, 5% CO2, humidified atmosphere) for 6 days (~144 hours).

6.2.4 CD14 purity check

6.2.4.1 Prepare the CD14-staining mix according to the table below. The MACS buffer should be cold.

| **Marker** | **Label** | **Dilution** | **µl per sample** |
| --- | --- | --- | --- |
| CD14 | PE | 1:50 | 8 |
| Live-dead | Aqua | 1:100 | 40 |
| MACS buffer | - | X | 352 |

6.2.4.2 Spin down the samples: 3 min, 300 x g, 4°C.

6.2.4.3 Discard the supernatant and stain one of the replicate PBMC samples with 100 µl of the CD14-staining mix. As a control, stain the other replicate PBMC sample with MACS buffer only (without the antibodies). The CD14+ and CD14- cells should be stained as well. Wrap the plate(s) in tin foil and put them in the dark at 4°C for 30 minutes.

| A1 | A2 | A3 | A4 |
| --- | --- | --- | --- |
| PBMC | PBMC | CD14+ | CD14- |
| unstained | **stained** | **stained** | **stained** |

6.2.4.4 Add 100 µl MACS buffer, re-suspend, and centrifuge at 300*g, 4°C for 3 minutes.

6.2.4.5 Remove the supernatant, add 100 µl MACS buffer to the wells, re-suspend, and centrifuge at 300*g, 4°C for 3 minutes.

6.2.4.6 Prepare 1 ml of cellFIX solution by adding 50 µl cellFIX to 950 µl MACS buffer. Remove the supernatant and re-suspend the cells in 200 µl cellFIX solution. Keep the plate at 4°C until measurement in the FACS Canto. For practical purposes, this measurement is done the next day.

6.2.4.7 Acquire the data on the FACS Canto II using the settings:

6.2.4.7.1 FSC 250*

6.2.4.7.2 SSC 400*

6.2.4.7.3 PE** 488 nm laser (blue) 585/42 filter

6.2.4.7.4 Aqua** 405 nm laser (violet) 510/50 filter

* for doublet discrimination, record both area and height

** only record area

6.2.4.7.5 Settings: sample flow rate 1.5 µl/sec; sample volume 170 µl; mixing volume 70 µl; mixing speed 180 µl; number of mixes 3; washing volume 800 µl.

6.2.4.7.6 Compensations: these should be set using beads and DC made on a population of 50% living cells and 50% dead cells. To obtain dead cells, heat-shock the living cells.

6.2.4.7.7 Gating:


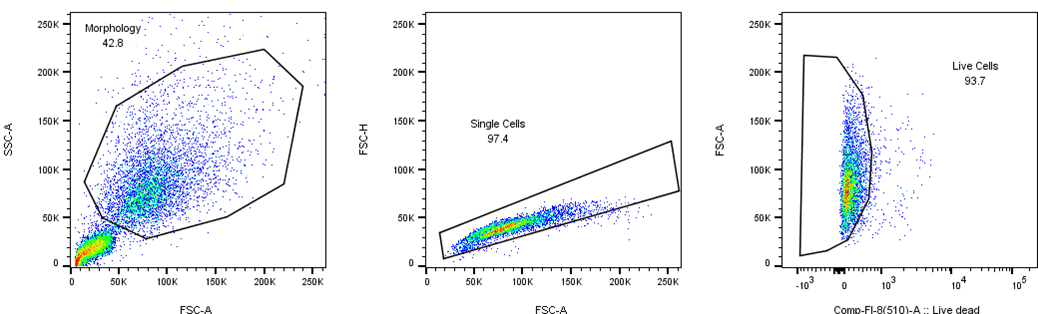


6.2.4.7.7.1 Gate based on the morphology of the cells (see graph on the left). Set the gating such that the lower left corner (<50K FSC-A (X-axis) and <50 K-SSC-A (Y-axis)) is excluded.

6.2.4.7.7.2 Within the cell population gated under (1), gate the single cells (see middle graph). In the FSC-A (X-axis) vs. FSC-H (Y-axis plot), doublet cells form a population below the diagonal.

6.2.4.7.7.3 Within the cell population gated under (2), gate the live cells (see graph on the right). In the live-dead staining (X-axis) vs. FSC-A (right axis), the dead cells scatter to the right.

6.2.5 Exposure to NMP:

6.2.5.1 Previous work should have resulted in a concentration range to be tested.

6.2.5.2 Previous work should have resulted in sufficient characterization of a similarly prepared dispersion.

6.2.5.3 Before preparing the concentration series of NMP(s), check the cells under the reversing microscope.

6.2.5.4 In the flow cabinet, prepare the 2-fold dilution series of NMPs as follows (see next page). NOTE 1: The scheme depicted below takes 1.28 mg/ml NMP as a suspension to start with; of course, any other concentration can be taken as start*. NOTE 2: In general, final concentrations in the order of 100 µg/ml are the highest concentrations tested in *in vitro* studies. The example on the next page uses 128 µg/ml as highest concentration.

First, calculate the volume needed for each concentration (in this case, each dose requires 120 µl).

6.2.5.4.1 Label seven tubes for ultrapure water.

6.2.5.4.2 Add 60 µl ultrapure water to each tube.

6.2.5.4.3 Sonicate the NMPs in a sonication bath at room temperature for 5 min.

6.2.5.4.4 In the flow cabinet, take 60 µl from the NMP suspension (of 1.28 mg/ml) and add it to a tube containing 60 µl ultrapure water (U) to obtain a 0.64 mg/ml suspension.

6.2.5.4.5 Mix by pipetting up and down.

6.2.5.4.6 Take 60 µl from the 0.64 mg/ml tube and add it to a tube containing 60 µl ultrapure water (U) to obtain a 0.32 mg/ml suspension.

6.2.5.4.7 Proceed until a 0.02 mg/ml suspension is obtained. It is not necessary to change the tip of the pipette.


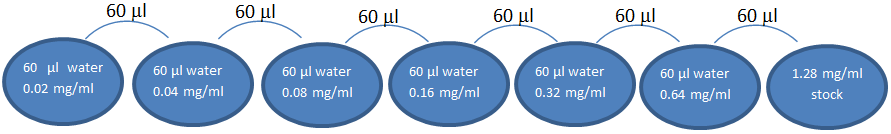


6.2.6 Exposure of immature DC

6.2.6.1 Prepare pre-dilutions of NMP and controls (e.g. LPS (TLR4 agonist; positive control), R848 (TLR7/8 agonist; positive control) and medium (negative control)) in PBS. These pre-dilutions should be 10x the concentration of the intended final concentration.

The FINAL concentration of LPS is 100 ng/ml; the FINAL concentration of R848 is 5 µg/ml.

6.2.6.2 Freshly prepare **exposure** medium (see before).

6.2.6.3 Gently remove 750 µl supernatant from the side of the wells containing the immature DC, leaving approximately 250 µl in the wells.

6.2.6.4 Mix 700 µl **exposure** medium with 107.7 µl pre-diluted NMP or control (of 10x the intended final concentration). Add 750 µl to the well containing the immature DC. Therefore, the final volume in the well is 1 ml.

6.2.6.5 Package the plate(s) with transparent film to prevent evaporation of medium. Put them in the incubator (37°C, 5% CO2, humidified atmosphere) for 48 hours.

6.2.7 Harvest and FACS analysis of DC

6.2.7.1 Gently dislodge the adherent cells from the plate with a (clean) plunger of a 1 ml syringe.

6.2.7.2 Rinse the well using a (clean) 1 ml pipette. Suck up all of the suspension and transfer it to a 50 ml tube on ice.

**NB: from here on, keep cells cold at all times!**

6.2.7.3 Add 0.5 ml cold PBS (4°C) to the emptied wells and incubate for a few minutes. You may continue with the next wells in the meantime.

6.2.7.4 Use a (clean) 1 ml pipette to rinse the well, suck up all of the suspension and transfer it to the dedicated 50 ml tube on ice.

6.2.7.5 Spin down the tubes (4°C, 300 x g, 8 min; “acceleration” 9, “brake” 5). Aliquot the supernatants for ELISA (e.g. 6x 125 µl aliquots or 4x 150µl aliquots) and freeze them at -20°C or -80°C.

6.2.7.6 Re-suspend the pellet and divide the cell suspension over two wells on two separate V-bottom 96- well plates*.

*The number of plates equals the number of staining panels.

6.2.8 Washing and staining cells for FACS analysis

6.2.8.1 Prepare the staining panels. Keep them cold and in the dark until further use.

| **Panel 1** |  |  |  |
| --- | --- | --- | --- |
| **Marker** | **Label** | **dilution** | **µl** |
| CD80 | FITC | 1:40 | 120 |
| CD14 | PE | 1:50 | 96 |
| PD-L1 | APC | 1:400 | 12 |
| HLA-DR | Pacific Blue | 1:1600 | 3 |
| Live/dead | Aqua | 1:1000 | 4.8 |
| Buffer | - |  | 4564.2 |

Total volume 4.8 ml

| **Panel 2** |  |  |  |
| --- | --- | --- | --- |
| **Marker** | **Label** | **dilution** | **µl** |
| CD83 | FITC | 1:40 | 120 |
| CD40 | PE | 1:20 | 240 |
| DC-SIGN | APC | 1:200 | 24 |
| CD86 | Pacific Blue | 1:800 | 6 |
| Live/dead | Aqua | 1:1000 | 4.8 |
| Buffer | - |  | 4405.2 |

Total volume 4.8 ml

6.2.8.2 Add 100 µl cold MACS buffer to the wells containing the cell suspensions and spin down the plates (4°C, 300 x g, 3 min; “acceleration” 9, “brake” 7).

6.2.8.3 Flick the plates over the sink to remove the supernatant and re-suspend the cells in 100 µl cold MACS. Spin down the plates.

6.2.8.4 Remove the supernatant (again by flicking over sink) and re-suspend the unstained controls with 100 µl cold MACS. Re-suspend the remaining wells with 100 µl of the prepared staining panels (one panel per plate).

6.2.8.5 Wrap the plates in tin foil and incubate in fridge (4°C) for 30 minutes.

6.2.8.6 Add 100 µl MACS buffer to all wells and spin down the plates.

6.2.8.7 Flick the plates over the sink to remove the supernatant and wash twice with 100 µl MACS buffer (re-suspend, spin down, remove supernatants; repeat).

6.2.8.8. Re-suspend the cells in 200 µl MACS buffer, wrap in tinfoil, and store cold (4°C) and in the dark until further use.

6.2.9 FACS Canto II settings for DC

6.2.9.1 This SOP does not include a description on the use of a FACS Canto II or in fact any other FACS machine. Only issues specific for FACS analysis of DC are discussed here.

6.2.9.2 Obtain appropriate settings is discussed in “Part 2. Rules Used to Create Application Settings”, which starts at page 6 of the brochure “Standardizing Application Setup Across Multiple Flow Cytometers Using BD FACSDiva™ Version 6 Software” by Becton Dickinson. This brochure should accompany the present SOP.

6.2.9.3 DC are auto-fluorescent cells; therefore, the settings of the FACS have to be adjusted to DC. These settings can be stored on the FACS machine and retrieved when measuring DC. BD Biosciences has developed a process in which application settings can be systematically created using three simple rules derived from key CS&T baseline information for each detector on the cytometer. The following rules are used to create the application settings: Rule 1. For each detector, determine a minimum PMT voltage setting for the appropriate unstained cells of interest or representative particles. The minimum acceptable voltage for each detector is set so that the resulting rSD of the unstained cells is approximately 2.5 times the rSDEN. This ensures that the electronic noise does not interfere with the measurements at the low end of the scale (see Formula 1).


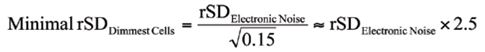


Reference (see page 6):

https://www.bdbiosciences.com/documents/BD_FACSDiva_Stndrd_App_Setup_TechBulletin.pdf

When this has been completed, a series with single colourings is done (on BD compensation beads) to determine the spectral overlap and thereby make compensation settings. The method is in the PDF that can be found at https://www.bdbiosciences.com/documents/facsdivav6_canto_quickrefguide_hts.pdf

With these single colourings, all channels in the device are represented. After completing these compensation checks, the settings are saved and can be retrieved by each user when performing a new measurement.

It is possible to slightly adjust the compensations after the measurement. This can be done during the analysis in FlowJo or possibly in FACS Diva itself. This is common practice in flow cytometry. Whether this needs to be done depends on the type of fluorochrome that was used or the combination of some fluorochromes.

6.2.9.4 The Canto is calibrated weekly (by dedicated RIVM staff) and maintained annually (by BD). If a new CS & T baseline has been required during maintenance, the settings must be re-created. Most often, this does not apply to a limited series of tests.

6.2.9.5 Acquire the data on the FACS Canto II using the settings:

6.2.9.5.1 FCS 150*

6.2.9.5.2 SSC 350*

6.2.9.5.3 FITC** 488 nm laser (blue) 530/30 filter

6.2.9.5.4 PE** 488 nm laser (blue) 585/42 filter

6.2.9.5.5 APC** 633 nm laser (red) 660/20 filter

6.2.9.5.6 Pacific blue** 405 nm laser (violet) 450/50 filter

6.2.9.5.7 Aqua** 405nm laser (violet) 510/50 filter

*for doublet discrimination, record both area and height

** record only area

6.2.9.6 Settings: sample flow rate 3 µl/sec; sample volume 170 µl; mixing volume 70 µl; mixing speed 180 µl; number of mixes 3; washing volume 800 µl.

6.2.10 Cell viability measurement:

6.2.10.1 Check the cells under the reversing microscope.

6.2.10.2 Take WST-1 from –20 °C and thaw.

6.2.10.3 Put WST-1 and the cell culture plate in the flow cabinet. Add 20 µl WST-1 to each well. Preferably, use a multi-channel pipette. Do not touch the medium with the pipette tips; it is not necessary to change the pipette tips.

6.2.10.4 Put the plates in the incubator. Incubate the cells for 1, 2 and 3 hours. Measure the absorbance with a spectrometer at 440 nm (420-480 nm advised) versus a 620 nm reference. The absorbance should be 1.5-3.0; if this is not reached after 3 hours, measure again after 4 hours (so 1 hour later) and use those values.

6.2.10.5. Calculate the cell viability:

6.2.10.5.1 For each NMP concentration (including control without NMP) calculate the average absorbance value of sample with cells (N=4 test samples; blue square in Figure 1) and that without cells (N=2 interference samples; red square).

6.2.10.5.2 For each NMP concentration (including control without NMP), subtract the absorbance of the interference samples from those of the test samples.

6.2.10.5.3 Cell Viability = (absorbance test sample - absorbance interference sample / absorbance control - absorbance interference control) x 100%.

6.2.11 IL-10 and IL-12p40 ELISA

It is advised to purchase ELISA kits, not antibody pairs. It is not necessary to purchase kits with coated plates, or kits with plates included. The ELISA can be performed according to the manufacturer’s instructions. Nunc Maxisorp plates perform well for a wide range of ELISA’s.

## Definition and Equation of the Measurand

## Statistical Data Evaluation

## Reporting of the Results

The most straightforward way of reporting the results on cell surface marker expression (FACS data) is the fold-induction of marker expression, per surface marker and per NMP concentration. Similarly, the most straightforward way of reporting the results on cytokine production (ELISA data) is the fold-induction of production, per cytokine and per NMP concentration.

Although each surface marker and each cytokine have a very well-known biological function, exposure effects on specific (sets of) of surface markers and cytokines do not have direct meaning of the biological effects of the NMP. Rather, upregulation (or downregulation in case of DC-SIGN) of several cell surface markers and cytokines provide a general indication of DC maturation.

# Potential Pitfalls

The most important source of variation is the inter-donor variation. It is therefore strongly recommended to perform the assay at the very least using 3 but preferably 5 donors. If equivocal are obtained, another 5 donors should be tested. Data presented should be a representative result obtained from single donor, with at least 2 other donors showing the similar data.

A second source of variation is the inter-operator variability. Minor inter-operator differences in cell handling may cause variability. It is therefore advised that a single technician performs the assay on all donors, or - should inter-operator variability be the subject of study - each technician should perform 3 or 5 experiments.

Differences in temperature, light/dark, and vibrations when performing the assay should be minimized.

# Quality Control and Acceptance Criteria

# Health and Safety Warnings, Cautions and Waste Treatment

# Abbreviations

# References

Banchereau J, Steinman RM. Dendritic cells and the control of immunity. Nature 1998;392:245–52.

Chiodo F, Marradi M, Park J, Ram AF, Penadés S, van Die I, Tefsen B. Galactofuranose-coated gold nanoparticles elicit a pro-inflammatory response in human monocyte-derived dendritic cells and are recognized by DC-SIGN. ACS Chem Biol 2014;9:383-9.

Fernández TD, Pearson JR, Leal MP, Torres MJ, Blanca M, Mayorga C, Le Guével X. Intracellular accumulation and immunological properties of fluorescent gold nanoclusters in human dendritic cells. Biomaterials 2015;43:1-12.

Satpathy AT, Wu X, Albring JC, Murphy KM. Re(de)fining the dendritic cell lineage. Nat Immunol 2012;13:1145-54.

# Annex

List of antibodies

| APC-CD209 | APC Mouse Anti-Human CD209 Clone DCN46 (RUO) | BD | 551545 | 100 tests |
| --- | --- | --- | --- | --- |
| APC-CD274 | CD274 (PD-L1, B7-H1) Monoclonal Antibody (MIH1), APC | eBioscience | 17-5983-42 | 100 tests |
| Aqua-L/D fixable | LIVE/DEAD® Fixable Aqua Dead Cell Stain Kit, for 405 nm excitation | Invitrogen | L34957 | 200 assays |
| FITC-CD80 | FITC Mouse Anti-Human CD80 Clone L307.4 (a.k.a. L307) (RUO) | BD | 557226 | 100 tests |
| FITC-CD83 | BD Pharmingen™ FITC Mouse Anti-Human CD83 | BD | 556910 | 100 tests |
| Pacific Blue-CD86 | Pacific Blue™ anti-human CD86 Antibody | Biolegend | 305417 | 25 µg |
| Pacific Blue-HLA-DR | Pacific Blue™ anti-human HLA-DR Antibody | Biolegend | 307623 | 25 µg |
| PE-CD14 | BD Pharmingen™ PE Mouse Anti-Human CD14 | BD | 555398 | 100 tests |
| PE-CD40 | BD Pharmingen™ PE Mouse Anti-Human CD40 | BD | 555589 | 100 tests |
